# Loss of C1q alters the auditory brainstem response

**DOI:** 10.3389/fncel.2024.1464670

**Published:** 2024-10-02

**Authors:** Sima M. Chokr, Ashley Bui-Tran, Karina S. Cramer

**Affiliations:** Department of Neurobiology and Behavior, University of California Irvine, Irvine, CA, United States

**Keywords:** auditory brainstem, microglia, medial nucleus of the trapezoid body (MNTB), neural development, complement

## Abstract

Neural circuits in the auditory brainstem compute interaural time and intensity differences used to determine the locations of sound sources. These circuits display features that are specialized for these functions. The projection from the ventral cochlear nucleus (VCN) to the medial nucleus of the trapezoid (MNTB) body travels along highly myelinated fibers and terminates in the calyx of Held. This monoinnervating synapse emerges during development as multiple inputs are eliminated. We previously demonstrated that elimination of microglia with a colony stimulating factor-1 inhibitor results in impaired synaptic pruning so that multiple calyceal terminals reside on principal cells of MNTB. This inhibitor also resulted in impaired auditory brainstem responses (ABRs), with elevated thresholds and increased peak latencies. Loss of the microglial fractalkine receptor, CX3CR1, decreased peak latencies in the ABR. The mechanisms underlying these effects are not known. One prominent microglial signaling pathway involved in synaptic pruning and plasticity during development and aging is the C1q-initiated compliment cascade. Here we investigated the classical complement pathway initiator, C1q, in auditory brainstem maturation. We found that C1q expression is detected in the MNTB by the first postnatal week. C1q levels increased with age and were detected within microglia and surrounding the soma of MNTB principal neurons. Loss of C1q did not affect microglia-dependent calyceal pruning. Excitatory and inhibitory synaptic markers in the MNTB and LSO were not altered with C1q deletion. ABRs showed that C1q KO mice had normal hearing thresholds but shortened peak latencies. Altogether this study uncovers the developmental time frame of C1q expression in the sound localization pathway and shows a subtle functional consequence of C1q knockdown.

## Introduction

Specialized auditory circuits that allow us to process and localize sound with precision rely on carefully orchestrated neurodevelopmental mechanisms. Neural circuit assembly requires axon guidance, synapse strengthening and pruning, and neuroglial communication. Circuit precision is heavily linked with the correct number of synapses, which is ensured through synapse tagging, glial cell contact, and engulfment of the unwanted synaptic connections. During auditory circuit development, synaptic pruning is selective, ensuring robust and accurate means to process and react to a given stimulus. Mechanisms by which high frequency sound sources are localized with precision include a thickly myelinated contralateral connection between the ventral cochlear nucleus (VCN) and the medial nucleus of the trapezoid body (MNTB). This enveloping synapse is referred to as the calyx of Held. The MNTB is a relay nucleus that provides inhibitory input to the lateral superior olive (LSO) which simultaneously receives excitatory input from spherical bushy cells from the ipsilateral VCN. These bilateral inputs result in an excitatory/inhibitory (E/I) ratio that aids in localization of sound sources (Boudreau and Tsuchitani, 1968; Gjoni et al., 2018; Grothe et al., 2010; Karcz et al., 2011; Magnusson et al., 2008; Tollin, 2003).

During development, multiple protocalyces, or immature calyces, are eliminated resulting in a single calyx innervating a single MNTB cell (Hoffpauir et al., 2006; Holcomb et al., 2013; Morest, 1969; Rodríguez-Contreras et al., 2008; Sätzler et al., 2002). Young, immature calyces are morphologically and electrophysiologically distinct from more mature and elaborate calyces (Hoffpauir et al., 2006; Rodríguez-Contreras et al., 2008; Sierksma et al., 2017). Presumably, the protcalyces that display weaker activity are detected and eliminated. However, this mechanism has yet to be unraveled (Kronander et al., 2019; Sierksma et al., 2020; Sierksma et al., 2017). Some developmental studies have pointed to microglia, the brain’s immune cells, as potential candidates for synaptic pruning and circuit formation (Bilimoria and Stevens, 2015; Erblich et al., 2011; Favuzzi et al., 2021; Hoshiko et al., 2012; Kettenmann et al., 2013; Matcovitch-Natan et al., 2016; Milinkeviciute et al., 2019; Paolicelli et al., 2011; Schafer et al., 2012). In the brainstem, loss of microglia through pharmacological inhibition of the colony stimulating factor 1 receptor (CSF1R), essential for microglial survival and proliferation, impairs calyceal elimination, resulting in higher instances of polyinnervated MNTB cells in mature animals (Milinkeviciute et al., 2019). Treatment cessation and microglial return restores the 1:1 synapse-cell ratio (Milinkeviciute et al., 2021b). Functionally, loss of microglia leads to auditory brainstem response (ABR) deficits, as shown by higher hearing thresholds and increased peak latencies (Milinkeviciute et al., 2021b). Microglial repopulation largely rescues these functional deficits as well (Milinkeviciute et al., 2021b). Long-term CSF1R inhibition prevents pruning recovery, elevates inhibitory protein levels, and sustains and worsens ABR threshold and latency deficits (Chokr et al., 2022). Investigation of a major signaling pathway, through the microglial fractalkine receptor (CX3CR1), revealed that congenital depletion of CX3CR1 does not affect calyceal pruning. However, CX3CR1 mutants have decreased ABR peak latencies, lack tonotopic size gradients in the MNTB, and do not display age-related decrease of inhibitory proteins, suggesting defects in inhibitory synaptic pruning (Milinkeviciute et al., 2021a). Together, these studies support a role for microglia in the formation of sound localization circuitry. However, our understanding of the mechanisms by which microglia regulate pruning and circuit maturity remains deficient.

Previous studies have demonstrated that that synapses are removed during neural development through microglia-secreted complement protein C1q-tagging and opsonization (Cong et al., 2022; Fonseca et al., 2017; Fonseca et al., 2004; Presumey et al., 2017; Stevens et al., 2007). The complement system is traditionally recognized for its role in immune defense and inflammation but in the brain has multifaceted functions in neurodevelopment and degeneration (Zhang et al., 2023). C1q is the initiator protein for the classical complement cascade. Once bound to pathogen or debris, a protease cascade is triggered where either complement protein C3 leads to macrophage/microglia-mediated excision or C3 triggers the terminal activation of the complement pathway which leads to lysis (Schafer et al., 2012; Stevens et al., 2007; Zhang et al., 2023). C1q is known as an “eat me” signal for synapse elimination, which in the context of neural development is pivotal to ensure circuit accuracy and achievement of the E/I balance (Dejanovic et al., 2022). However, the effects of this signal are heterogeneous throughout brain regions and time frames. In the visual cortex, loss of C1q does not affect synapse density or microglial phagocytosis (Cong et al., 2022; Zhang et al., 2023). In an epilepsy model, C1q has a neuroprotective role and prevents synaptic pruning, its consequences appearing in seizures from improper E/I ratios (Chu et al., 2010).

Our understanding of C1q in auditory system development is lacking, despite implications on regulation of E/I ratios, an essential component for accurate auditory processing. In the cochlea, complement C1q Like 1 (C1QL1), a secreted component of C1Q-related protein, is expressed in adult inner and outer hair cells (Liu et al., 2018; Liu et al., 2014; Qi et al., 2021). C1QL1 is expressed in outer hair cells in a tonotopic gradient along the cochlea (Biswas et al., 2021). Loss of C1QL1 reduces the number of nerve fibers innervating hair cells, progresses loss of outer hair cells, leads to increased hearing thresholds, and increases peak 1 latency in the ABR (Qi et al., 2021).

The role of C1q in auditory brainstem development has not been investigated. Here, we studied the effects of C1q in development of auditory brainstem circuitry. We used a C1q knockout (KO) model to test whether loss of C1q affects calyceal pruning levels, synaptic protein expression, and auditory function. We found that, like microglial markers, C1q expression is detected as early as postnatal day (P) 8 and increases by P14, just after hearing onset. C1q expression was detected both in a colocalized fashion with vesicular glutamate transporter 1/2 (VGLUT1/2) protein surrounding MNTB cells and in a net-like pattern throughout the MNTB independent of VGLUT1/2 puncta. Super-resolution imaging revealed that C1q surrounds MNTB neurons in close proximity to VGLUT1/2-positive calyceal synapses. We also found that C1q is present within microglial somata and processes. Loss of C1q did not affect calyceal pruning or calyx size. Neither excitatory nor inhibitory synaptic protein levels were affected in the MNTB or LSO. Auditory brainstem responses showed that C1q KO mice had normal hearing thresholds, but faster peak latencies along the ascending auditory pathway. These data support that C1q regulates some functional aspects of auditory circuit development, but it is not responsible for regulating E/I synapses in the superior olivary complex.

## Methods

### Animals

We used C57/BL6 mice of both sexes at postnatal day (P) 8 (*n* = 12), P14 (*n* = 18), and P28 (*n* = 27). To investigate the effects of C1q depletion, we used (*n* = 41, P28) C1qa knock-out mice (C1q KO) (Jackson lab ID: 031675) (Fonseca et al., 2017). C1q KO was routinely confirmed during colony maintenance with qPCR (Fonseca et al., 2017). For C1q localization studies in relation to microglia, we used CX3CR1^+/GFP^ mice at P8 (*n* = 3) and P14 (*n* = 10) (Jung et al., 2000). All animal procedures were performed in accordance with the Institutional Animal Care and Use Committee at the University of California, Irvine (UCI). Mice were reared in a standard day/light cycle and received food and water *ad libitum*. Mice were housed in groups with no more than 5 adult mice per cage. Litters remained with the nursing dam until at least P21.

### Neuronal tracing

Calyceal pruning was assessed using a dye-insertion method as previously described (Chokr et al., 2022; Milinkeviciute et al., 2021a; Milinkeviciute et al., 2021b; Milinkeviciute et al., 2019). Briefly, P28 mice were transcardially perfused with oxygenated artificial cerebrospinal fluid (aCSF; 130 mM NaCl, 3 mM KCl, 1.2 mM KH2PO4, 20 mM NaHCO3, 3 mM HEPES, 10 mM glucose, 2 mM CaCl2, 1.3 mM MgSO4 infused with 95% O2 and 5% CO2). Brains were extracted and transferred to oxygenated solution for approximately 30 min and were then transferred to an aCSF filled petri dish. A glass micropipette was filled with rhodamine dextran (RDA; MW 3000, Invitrogen; in solution of 6.35% RDA with 0.4% Triton-X100 in PBS). The pipette was inserted in the midline to aim for the ventral acoustic stria and RDA was electroporated at 5 pulses/s at 55 V for 50 ms using an Electro Square Porator (ECM830; BTX). These pulses result in a sparse dye labeling of globular bushy cell axons and their calyceal terminals. Brains were transferred back to the oxygenated aCSF and the dye was allowed to travel for approximately 2 h. The tissue was then transferred to 4% paraformaldehyde solution refrigerated overnight, and then transferred to a 30% sucrose solution in 0.1 M PBS until cryosectioning. Brainstems were coronally cryosectioned at 20 μm in a series of 5 alternating slides. Tissue sections containing RDA-labeled calyces were immunohistochemically stained for vesicular glutamate transporter 1/2 (VGLUT1/2).

### Determination of mono- or polyinnervation

RDA-filled calyces were imaged using confocal microscopy (Leica SP8, 40X oil objective, zoom: 2.37, pinhole: 0.43). *Z*-stack images of Nissl, RDA, and VGLUT1/2 were acquired at a resolution of 1024 × 1024, with a *z*-step size of 0.32 μm. Gain and offset were adjusted accordingly if the intensity was noticeably different in comparison with other sections on the same slide.

Image stacks were reconstructed and analyzed using Imaris software (version 10.0; Bitplane). Calyces were assessed for quality by ensuring that each calyx was fully visible within the *z*-stack and had a visible preterminal axon segment. Calyces were reconstructed using the surface module with 0.125–0.3 surface detail to capture an accurate depiction of calyx processes shape. Calyceal surface area and volume were measured.

Mono- or polyinnervation status of the MNTB neuron was determined by visualizing the RDA-filled calyx with a Nissl-stained MNTB cell. MNTB cells contacted by an RDA-filled calyx, without VGLUT1/2 immunolabeling surrounding non-calyceal spaces, were designated as monoinnervated. Cells were classified as polyinnervated if VGLUT1/2 labeling surrounded non-calyceal spaces around the cell. Neurons were only considered polyinnervated if the non-calyceal VGLUT1/2 immunolabel surrounded ~25% or more of the MNTB cell (Milinkeviciute et al., 2019). We compared the percentage of mono- versus polyinnervated neurons in control and C1q KO mice.

### Immunolabeling

Brain sections were stained for complement protein C1q and vesicular glutamate transporter 1/2 (VGLUT1/2), vesicular glutamate transporter 2 (VGLUT2), or glycine transporter 2 (GLYT2). Mounted brain sections were outlined with hydrophobic PAP pen barrier and kept on a slide warmer for 10 min until dry. Sections were rinsed with 0.1 M phosphate buffer saline (PBS) for 10 min then incubated in 0.1% sodium dodecyl sulfate in PBS for antigen retrieval (5 min) and rinsed with PBS. Next, sections were incubated in blocking solution containing 5% normal goat serum (NGS; Vector Laboratories S-1000) and 0.3% Triton X-100 (Acros 9,002-93-1) in 0.1 M PBS for 1 h at room temperature, followed by overnight incubation in blocking solution containing the vesicular glutamate transporter 1/2 (VGLUT1/2), VGLUT2 antibody (1,200 dilution, Synaptic Systems, 135,503 or 135,418), or GLYT2 antibody (1,200 dilution, Synaptic Systems, 272,003). Tissue probed for C1q was incubated in anti-mouse C1q (rabbit monoclonal, clone 27.1) culture supernatant as described in Fonseca et al. (2017), Li et al. (2008), and Stephan et al. (2013) with 0.3% Triton X-100 and 0.5% bovine serum albumin. The tissue was then rinsed with PBS and incubated for 90 min in blocking buffer containing goat anti-rabbit tagged with an Alexa (Invitrogen) fluorophore (1,500 dilution, anti-rabbit 647, A21244, anti-rabbit 555, A21428 or anti-guinea pig A11073). Sections were then washed with PBS and incubated in blue fluorescent Nissl stain (NeuroTrace 435/455, Life Technologies N21479) diluted 1:200 in 0.3% Triton X-100 in 0.1 M PBS. Sections were rinsed with PBS and coverslipped with Glycergel mounting medium (Dako C0563). Statistical analyses for immunolabeling results are presented in Table 1.

**Table 1:** Immunolabeling analyses.

**C1q coverage across tonotopic axis**			
**2-way Anova**			
**Source of variation**	***p* value**	***F* (DFn, DFd)**	
Interaction	<0.0001	*F* (2, 66) = 45.67	
Age	0.1332	*F* (1, 66) = 2.311	
MNTB region	0.0089	*F* (2, 66) = 5.072	
**Šídák’s multiple comparisons test**	***p* value**	***t***	**DF**
P8:Medial vs. P8:Central	0.9995	0.8576	66
P8:Medial vs. P8:Lateral	<0.0001	5.702	66
P8:Medial vs. P14:Medial	<0.0001	5.52	66
P8:Central vs. P8:Lateral	0.0001	4.844	66
P8:Central vs. P14:Central	>0.9999	0.2072	66
P8:Lateral vs. P14:Lateral	<0.0001	7.946	66
P14:Medial vs. P14:Central	<0.0001	6.615	66
P14:Medial vs. P14:Lateral	<0.0001	9.169	66
P14:Central vs. P14:Lateral	0.1776	2.554	66
**Polyinnervation ratio**			
**Mann–Whitney test**	***p* value**		
WT vs. C1q KO	0.9365		
Descriptive statistics	WT	C1q KO	
Mean	0.2801	0.2133	
Std. Deviation	0.1564	0.03613	
Std. Error of Mean	0.07818	0.01616	
**Calyx surface area**			
**Mann–Whitney test**	***p* value**		
WT vs. C1q KO	0.6468		
Descriptive statistics	WT	C1q KO	
Mean	575.2	551.3	
Std. Deviation	225.5	234.4	
Std. Error of Mean	47.03	45.11	
**Calyx volume**			
**Mann–Whitney test**	***p* value**		
WT vs. C1q KO	0.4252		
Descriptive statistics	WT	C1q KO	
Mean	240.9	211	
Std. Deviation	116.5	109.2	
Std. Error of Mean	24.3	22.29	
**VGLUT 1/2 MNTB coverage total**			
**Mann–Whitney test**	***p* value**		
WT vs. C1q KO	0.1143		
Descriptive statistics	WT	C1q KO	
Mean	0.04362	0.08103	
Std. Deviation	0.01032	0.04266	
Std. Error of Mean	0.005159	0.01742	

**VGLUT1/2 MNTB coverage across tonotopic axis**			
**2-way Anova**			
**Source of variation**	***p* value**	***F* (DFn, DFd)**	
Interaction	0.4335	*F* (2, 24) = 0.8656	
Tonotopic region	0.0125	*F* (2, 24) = 5.291	
Genotype	0.0132	*F* (1, 24) = 7.159	
**Tukey’s multiple comparisons test**	***P* value**	***q***	**DF**
Medial			
WT vs. C1q KO	0.6248	0.7006	24
Central			
WT vs. C1q KO	0.0326	3.209	24
Lateral			
WT vs. C1q KO	0.0737	2.645	24
WT			
Medial vs. Central	0.4475	1.74	24
Medial vs. Lateral	0.6919	1.166	24
Central vs. Lateral	0.9135	0.5741	24
C1q KO			
Medial vs. Central	0.0052	4.935	24
Medial vs. Lateral	0.0451	3.601	24
Central vs. Lateral	0.619	1.334	24
**VGLUT1/2 LSO coverage total**			
**Mann–Whitney test**	***p* value**		
WT vs. C1q KO	0.1167		
Descriptive statistics	WT	C1q KO	
Mean	0.4606	0.4091	
Std. Deviation	0.03059	0.04696	
Std. Error of Mean	0.01766	0.01775	
**2-way Anova**			
**Source of variation**	***p* value**	***F* (DFn, DFd)**	
Interaction	0.5658	*F* (2, 16) = 0.5903	
Tonotopic region	<0.0001	*F* (1.574, 12.59) = 44.95	
Genotype	0.1138	*F* (1, 8) = 3.151	
**Tukey’s multiple comparisons test**	***p* value**	***q***	**DF**
Medial			
WT vs. C1q KO	0.8061	0.3622	6.23
Central			
WT vs. C1q KO	0.2362	2.019	3.497
Lateral			
WT vs. C1q KO	0.0248	3.909	7.913
WT			
Medial vs. Central	0.0067	23.11	2
Medial vs. Lateral	0.0853	6.26	2
Central vs. Lateral	0.1994	3.828	2
C1q KO			
Medial vs. Central	0.0004	11.95	6
Medial vs. Lateral	0.0946	3.62	6
Central vs. Lateral	0.0051	7.289	6
**GLYT2 MNTB coverage total**			
**Mann–Whitney test**	***p* value**		
WT vs. C1q KO	>0.9999		
Descriptive statistics	WT	C1q KO	
Mean	0.01765	0.02799	
Std. Deviation	0.01247	0.02849	
Std. Error of Mean	0.005577	0.01274	

**GLYT2 MNTB coverage across tonotopic axis**			
**2-way Anova**			
**Source of variation**	***p* value**	***F* (DFn, DFd)**	
Interaction	0.5303	*F* (2, 16) = 0.6602	
Tonotopic region	0.1395	*F* (1.962, 15.69) = 2.245	
Genotype	0.455	*F* (1, 8) = 0.6164	
**Tukey’s multiple comparisons test**	***p* value**	***q***	**DF**
Medial			
WT vs. C1q KO	0.4129	1.286	4.163
Central			
WT vs. C1q KO	0.4635	1.101	6.548
Lateral			
WT vs. C1q KO	0.8432	0.2904	6.934
WT			
Medial vs. Central	0.4304	1.954	4
Medial vs. Lateral	0.0597	4.756	4
Central vs. Lateral	0.9268	0.5303	4
C1q KO			
Medial vs. Central	0.4172	1.998	4
Medial vs. Lateral	0.966	0.3558	4
Central vs. Lateral	0.3427	2.272	4
**GLYT2 LSO coverage total**			
**Mann–Whitney test**	***p* value**		
WT vs. C1q KO	>0.9999		
Descriptive statistics	WT	C1q KO	
Mean	0.2994	0.294	
Std. Deviation	0.03702	0.05418	
Std. Error of Mean	0.01851	0.02423	

**GLYT2 LSO coverage across tonotopic axis**			
**2-way Anova**			
**Source of variation**	***p* value**	***F* (DFn, DFd)**	
Interaction	0.5518	*F* (2, 14) = 0.6206	
Tonotopic region	<0.0001	*F* (1.146, 8.025) = 50.73	
Genotype	0.7912	*F* (1, 7) = 0.07569	
**Tukey’s multiple comparisons test**	***p* value**	***q***	**DF**
Medial			
WT vs. C1q KO	0.7618	0.4466	6.676
Central			
WT vs. C1q KO	0.7159	0.5361	6.981
Lateral			
WT vs. C1q KO	0.4895	1.032	6.883
WT			
Medial vs. Central	0.0152	9.155	3
Medial vs. Lateral	0.25	2.884	3
Central vs. Lateral	0.0056	12.98	3
C1q KO			
Medial vs. Central	0.0687	4.538	4
Medial vs. Lateral	0.0917	4.103	4
Central vs. Lateral	0.0001	25.63	4

### Fluorescent imaging and analysis

Immunolabeled sections were visualized and imaged with a Zeiss Axioskop-2 microscope, an Axiocam camera, and Zen image analysis software. We analyzed the auditory nuclei on both sides of the midline. For each animal in our analysis, at least three sections were included per primary antibody stain. Images were exported and analyzed using FIJI imaging software. The corresponding Nissl image was used to guide outlines for the nuclei using the ROI function, and these outlines were then used to calculate percent coverage in the ROI of the channel containing the primary antibody stain as previously described (Milinkeviciute et al., 2021b; Milinkeviciute et al., 2019).

### Super-resolution microscopy

Super-resolution images of GFP-microglia, RDA-labeled calyces, and C1q staining were obtained with the ZEISS Elyra 7 with Lattice SIM^2^ microscope. Stained sections were imaged with a 63× oil immersion lens with 2.0 laser power settings. Following confirmation of C1q presence throughout each selected microglia, approximately 6–9 μm *Z*-stack images were obtained in 0.3 μm increments. These imaging settings led to a 60 nm resolution, allowing us to obtain accurate spatial settings of C1q protein relative to microglial somas and branches. Images were then processed through the Zeiss black software with SIM^2^ settings set to “Standard Fixed.” Once processed, images were converted to IMARIS compatible files. We used the IMARIS 10.0 software for background subtraction and representative image editing.

### Auditory brainstem responses

Auditory brainstem responses (ABRs) were tested on 7 control and 11 C1q KO mice of both sexes at P28. Mice used for ABRs were selected at random and used for synaptic protein assessment following collection. We used the same ABR recording methods as previously described (Chokr et al., 2022; Milinkeviciute et al., 2021b). Mice were anesthetized with an intramuscular injection of ketamine (75 mg/kg, KetaVed, VEDCO) and xylazine (15 mg/kg, AnaSed, NADA #139-236). Body temperature was maintained at 35°C via a far infrared warming pad (Kent Scientific, RT-0501) and sterile ocular lubricant (Puralube Vet Ointment, 006PHM02-1-8) was applied on the eyes. We inserted three pin electrodes subcutaneously with the positive, negative, and ground electrodes at the vertex, right cheek, and back near the right leg, respectively. The electrodes were connected to Tucker-Davis Technologies (TDT) RA4PA 4-channel Medusa amplifier, which was connected to a TDT RA16 Medusa Base Station. The ABR was performed in a sound-attenuating chamber (102 × 98 × 81 cm, Industrial Acoustics Company). Click and pure tone stimuli were generated using the TDT SigGen software version 4.4. Sound was presented with the TDT MF1 Multi-Function Speaker through an ear tube inserted in the animal’s left ear, with stimuli repeated 500 times at a rate of 21 stimuli per second. The stimuli were emitted using the TDT RP2.1 enhanced real time processor and the sound level was controlled with the TDT PA5 programmable attenuator. The recorded responses were amplified by the TDT SA1 stereo power amp and filtered through BioSig software version 4.4. Each sample ABR was recorded for 12 ms in response to 100 μs click or 3 ms pure tone stimuli (4, 8, 12, 16, 24, 32 kHz) and decreasing sound intensities (5 dB SPL steps from 80 to 10 dB SPL). An averaged response was computed at each sound level and was used for ABR analysis.

### ABR analysis

Averaged ABR recordings were assessed for hearing threshold, peak latency, interpeak latency, and peak amplitude. We defined hearing threshold as the lowest sound intensity at which peak I level (μV) was ≥4 standard deviations above the noise level (Bogaerts et al., 2009; Chokr et al., 2022; Milinkeviciute et al., 2021b). Peaks were manually detected and labeled by a blinded observer using BioSig, and data were exported for analysis. Peak latency was determined as the time from stimulus onset to the apex of the peak. Interpeak latency was calculated as the difference of absolute peak time between peaks I–II, II–III, III–IV, I–III, and I–IV. Peak amplitude was determined as the change in microvolts between the preceding trough and the apex of the subsequent peak. All ABR mean, standard error, and statistics are presented in Supplementary Table S1.

### Statistics

Multiple litters were used for each experimental group. Littermate controls were not used for experiments that included the congenital mutation as the breeders for C1q KO were mutants. Quantitative results are presented as the mean ± SEM. All statistical analyses were performed using Prism Software (v9.3.1; GraphPad Software). Comparisons between genotype or age group were made with a Welch’s *t* test, a Mann–Whitney test, or a two-way ANOVA with Sidak’s multiple comparisons test unless otherwise indicated. Statistical significance was accepted at *p* < 0.05. Details of statistical analyses are presented in Table 1 and Supplementary Table S1.

## Results

### C1q is present in MNTB during circuit refinement

Microglia appear in the auditory brainstem as early as P0 in the ventral cochlear nucleus (VCN) and by P6 in the medial nucleus of the trapezoid body (MNTB) (Dinh et al., 2014). Microglia are the primary source of C1q in the brain and C1q increases with age throughout the brain (Fonseca et al., 2017; Stephan et al., 2013). In other brain regions, C1q plays a developmental role in synapse protection, elimination, and ultimately circuit formation. Whether C1q is present in the brainstem during a period of circuit refinement was unknown. Here, we characterized C1q expression before and after hearing onset in wildtype mice.

**Figure 1:**
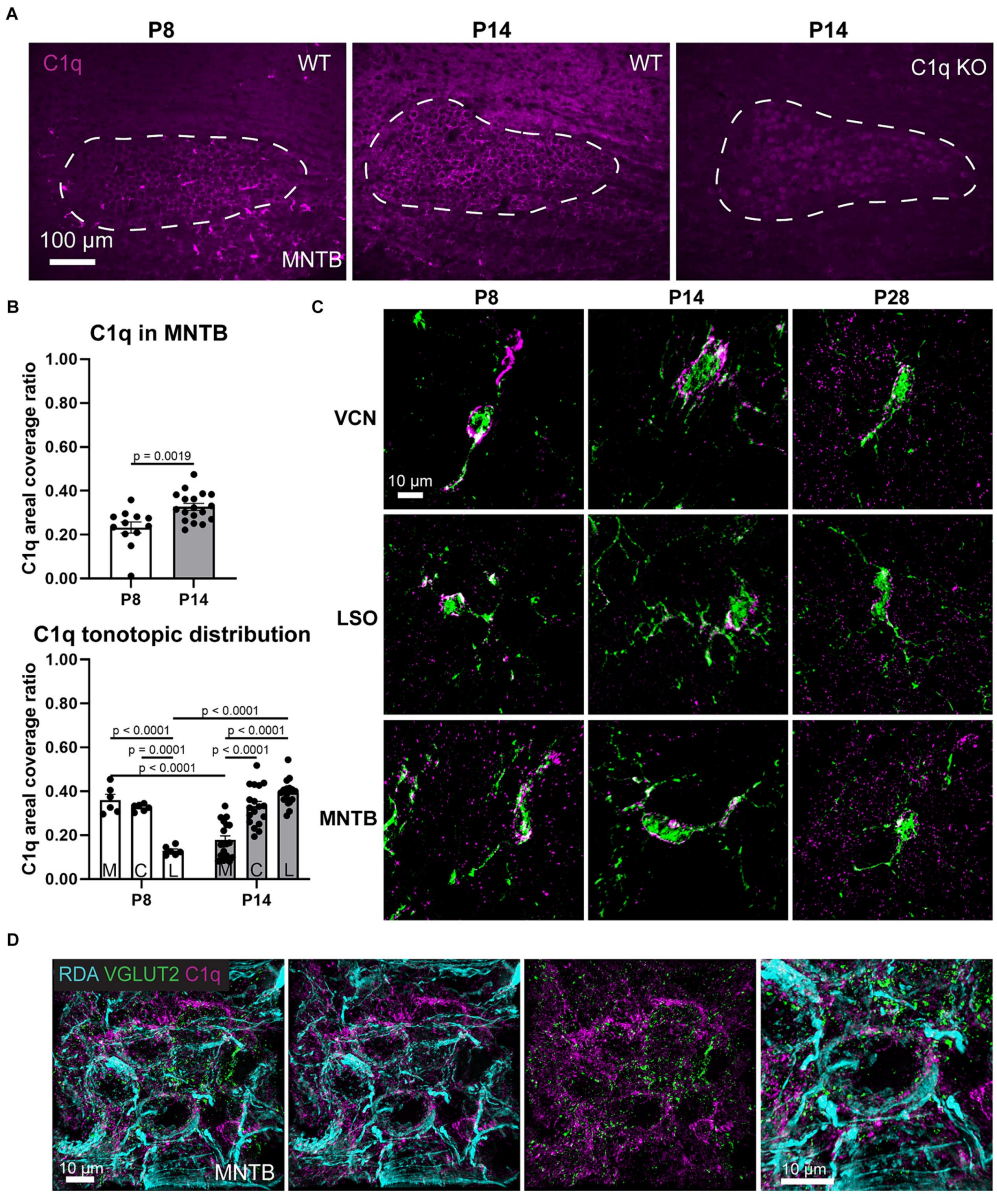
C1q is expressed in the developing auditory brainstem. **(A)** At P8, C1q (magenta) can be detected in the MNTB and appears in clusters resembling glia. P14 C1q expression appears dispersed throughout the MNTB. There was no immunoreactivity in C1q KO mice. **(B)** C1q levels significantly increase after hearing onset. At P8, C1q levels are higher in medial regions and at P14, C1q levels are higher in lateral regions. **(C)** Super-resolution images visualizing C1q expression (magenta) within GFP-microglia (green). C1q can be detected throughout the microglial somas and branches. In the mature animal (P28) C1q protein was also detected outside of microglial cells. **(D)** Rhodamine-dye labeled calyces (cyan) with C1q (magenta) and excitatory synapse marker VGLUT2 (green). C1q tags calyces and surrounds the non-calyceal regions around MNTB cells.

We assessed whether C1q is present during microglia-dependent circuit formation. At postnatal day (P) 8, C1q was expressed throughout the MNTB with an average areal coverage ratio of 0.2331 ± 0.02521 (*n* = 12) (Figures 1A,B). At P8, C1q surrounded MNTB cells and appeared in clusters resembling microglia (Figure 1A). At P14, C1q coverage ratios (0.3268 ± 0.0157, *n* = 18) significantly increased with age (*p* = 0.0019, Mann Whitney U = 37) and patterns appeared more uniformly diffuse (Figures 1A,B). Before hearing onset, C1q expression across the tonotopic axis significantly diminished in coverage from the medial (0.3594 ± 0.0013) and central (0.3037 ± 0.0223) regions to low frequency lateral regions (0.1193 ± 0.0103), respectively (Medial-Central *p* = 0.995, *t* = 0.8576, df = 66, Medial-Lateral *p* < 0.0001, *t* = 5.702, df = 66, Central-Lateral *p* = 0.0001, *t* = 4.844, df = 66) (Figure 1B). This expression gradient shifted at P14. C1q expression was significantly lower in medial regions (0.1761 ± 0.0019), remained comparable in central regions (0.3237 ± 0.0091) and increased in lateral regions (0.3829 ± 0.0097) (Medial-Central *p* < 0.0001, *t* = 6.615, df = 66, Medial-Lateral *p* < 0.0001, *t* = 2.554, df = 66, Central-Lateral *p* = 0.1776, *t* = 2.554, df = 66) (Figure 1B). We also found that these expression values differed with age, where medial C1q expression was significantly diminished after hearing onset (*p* < 0.0001, *t* = 5.520, df = 66) and lateral C1q expression was significantly elevated after hearing onset (*p* < 0.0001, *t* = 7.946, df = 66) (Figure 1B). C1q KO mice lacked any C1q expression in the brainstem (Figure 1A). Therefore, we determined that C1q is present in the MNTB during a period of microglia-dependent pruning and circuit refinement, and that tonotopic C1q expression patterns shift after hearing onset.

We further investigated C1q expression by obtaining super-resolution images of C1q before and after hearing onset, and in the mature animal. Microglia are the primary source of C1q in the brain but whether microglia express C1q during development was not known. At P8, we found that C1q is mostly present within microglial cells in the VCN, LSO, and MNTB, with remarkably less expression in the surrounding area (Figure 1C). In microglia with larger soma or less branching, indicating younger microglia, C1q levels appeared denser (Figure 1C). After hearing onset, C1q appeared present both within and outside of microglia, with higher density in microglia with larger somas (Figure 1C). Further, we found that at P28, C1q was present in close proximity to calyces visualized with rhodamine-dye (RDA) and VGLUT2 co-labeling (Figure 1D). These data indicated the anatomical location of C1q during MNTB development and lead us to investigate whether calyceal pruning involves C1q.

### C1q removal does not impair calyceal monoinnervation

We found that C1q levels in wildtype animals are elevated just after hearing onset, a period of microglia-dependent calyceal refinement. Therefore, we tested whether loss of microglial C1q tagging affects the establishment of calyceal monoinnervation of MNTB neurons. We sparsely labeled calyces using rhodamine dye (RDA) injections in the ventral acoustic stria and immunolabeled VGLUT1/2 on those sections to detect calyces in the MNTB. Principal cells that were almost exclusively surrounded by an RDA-labeled calyx were determined as monoinnervated, while cells surrounded by an RDA calyx and at least 25% VGLUT1/2 labeling were determined polyinnervated (see Materials and Methods). Confocal images were reconstructed and analyzed on IMARIS software.

**Figure 2:**
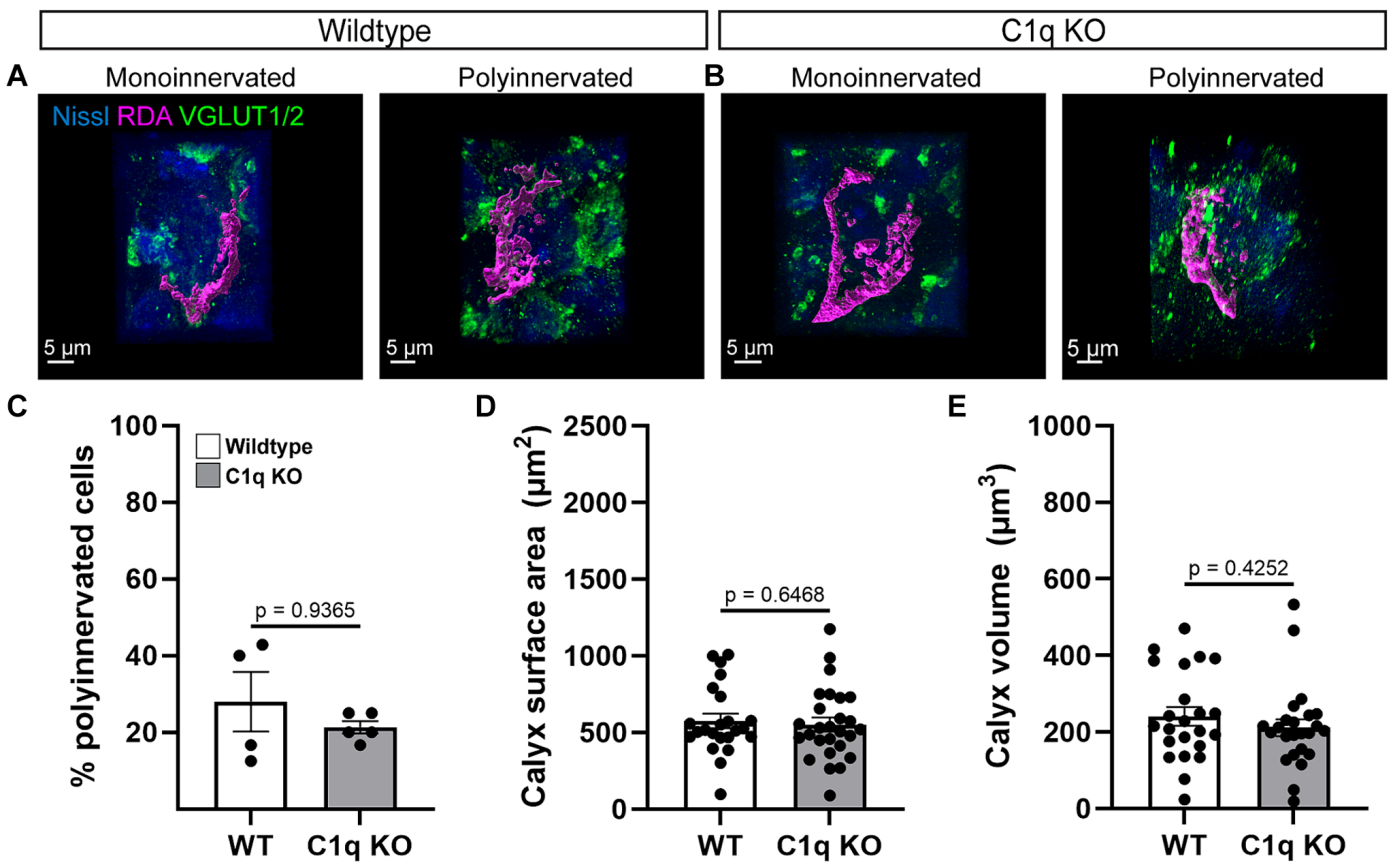
C1q deletion does not affect calyceal pruning. **(A,B)** Rhodamine-dye (RDA) labeled calyces (magenta) were 3D reconstructed and visualized with a Nissl (blue) and VGLUT1/2 (green) colabel. Mono- and polyinnervated cells were detected in both genotypes. **(C)** C1q deletion did not alter pruning. **(D)** Calyx surface areas in C1q KO were comparable with controls. **(E)** Calyx volumes in C1q KO were similar to control sizes.

We assessed calyces for innervation and size in 4 control and 5 C1q KO mice at P28. Both groups showed mono- and polyinnervated MNTB cells (Figures 2A,B). We determined the percentage of mono- vs. polyinnervated cells in each animal (WT 0.28 ± 0.07% of cells, C1q KO 0.21 ± 0.02% of cells) and found that C1q deletion does not affect calyceal elimination during development (*p* = 0.3790, *t* = 0.9289, df = 7, Welch’s *t*-test) (Figure 2C). Next, we measured the calyx surface area of RDA labeled calyces in WT (572.3 ± 47.03 μm^2^) and C1q KO (551.3 ± 45.11 μm^2^). We found that C1q elimination does not affect calyx surface area (*p* = 0.7154, *t* = 0.3667, df = 48, Welch’s *t*-test) (Figure 2D). Calyx volume was also assessed in both groups (WT 240.9 ± 24.30 μm^3^, C1q KO 211.0 ± 22.29 μm^3^). We did not see effects of C1q removal on calyx volume (*p* = 0.3688, *t* = 0.9079, df = 45, Welch’s *t*-test; Figure 2E). Altogether we found that removal of the microglial C1q signaling pathway did not affect the robust establishment of monoinnervation in the MNTB.

### Loss of C1q does not alter excitatory or inhibitory synaptic protein expression

**Figure 3:**
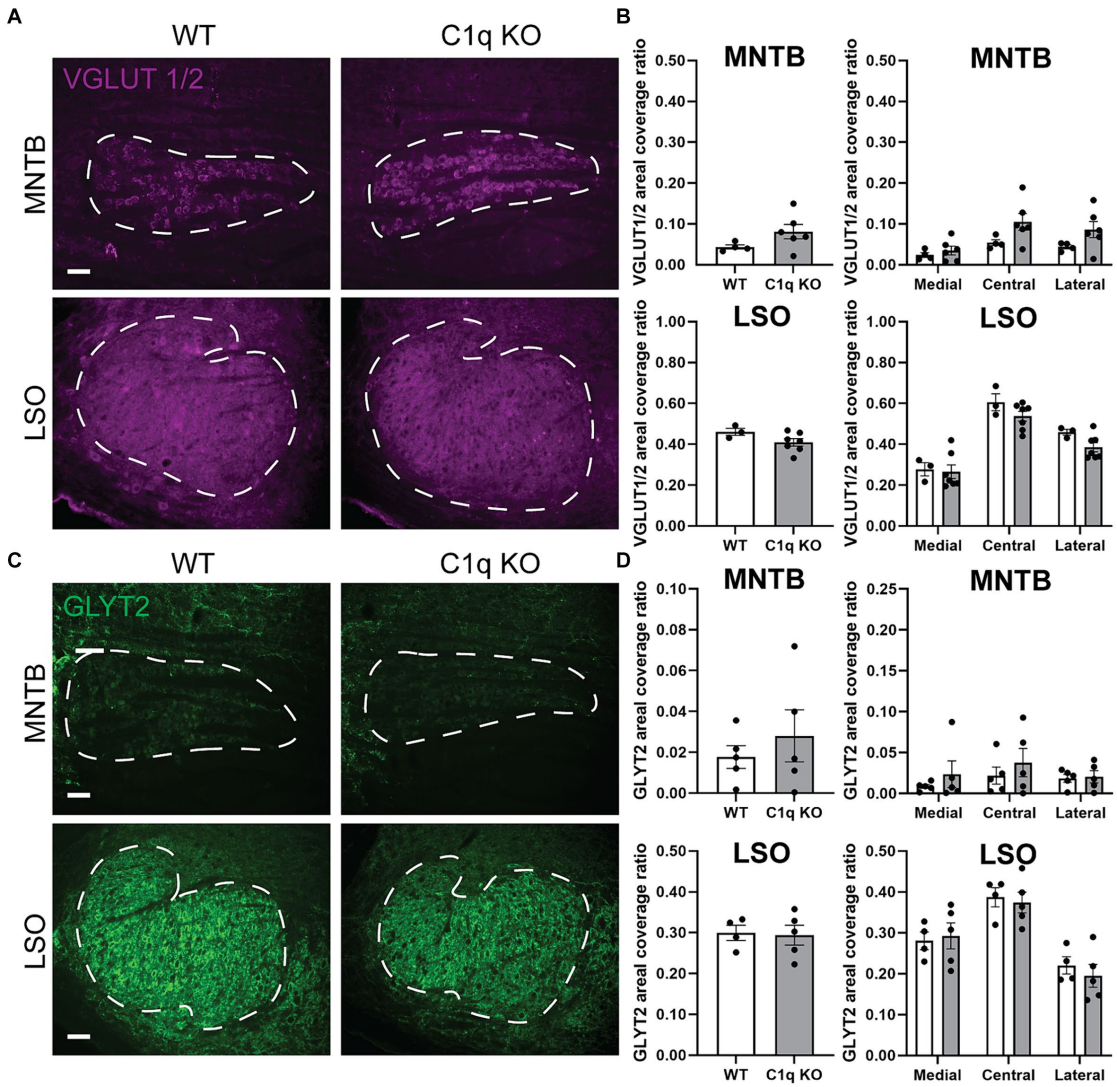
Excitatory and inhibitory presynaptic proteins are not altered with C1q knockout. **(A)** Immunolabeling of excitatory protein marker VGLUT1/2 (magenta) in WT and C1q KO mice in the MNTB and LSO, respectively. **(B)** C1q deletion did not alter VGLUT levels in the MNTB or LSO. **(C)** Immunolabeling of the inhibitory synapse marker GLYT2 (green) in WT and C1q KO mice in the MNTB and LSO, respectively. **(D)** GLYT2 levels were not affected by C1q knockout.

Pharmacological elimination of microglia disrupted excitatory calyceal pruning (Chokr et al., 2022; Milinkeviciute et al., 2019). Congenital elimination of CX3CR1 impaired inhibitory synapse elimination (Milinkeviciute et al., 2021a). In the lateral geniculate nucleus, loss of C1q leads to an abnormal E/I synapse ratio (Presumey et al., 2017; Stevens et al., 2007). However, some aspects of neural development and synaptic strengthening do not depend on C1q (Welsh et al., 2020). Therefore, we tested whether C1q KO mice exhibit an changes in excitatory and inhibitory presynaptic protein levels. We assessed excitatory protein expression by immunolabeling VGLUT1/2 puncta in coronal brainstem sections. In the MNTB, VGLUT1/2 areal coverage ratios were comparable between wildtype (0.04362 ± 0.01) and C1q KO (0.08103 ± 0.02) (*p* = 9.1143, Mann–Whitney test) (Figures 3A,B). VGLUT1/2 expression across the MNTB tonotopic showed a significant interaction between tonotopic region and genotype (*p* = 0.0147, 2-way ANOVA, *F* (2, 16) = 5.559) (Figure 3B). However, multiple comparison analysis did not indicate genotype differences in any of the regions (medial *p* = 0.7722, central *p* = 0.1612, lateral *p* = 0.2403) (Figure 3B). The LSO predominantly receives excitatory input from the ipsilateral VCN. We tested whether loss of C1q affects VGLUT1/2 expression in the LSO. We found that VGLUT1/2 levels were comparable between WT (0.4606 ± 0.017) and C1q KO mice (0.4091 ± 0.012) (*p* = 0.1167, Mann–Whitney test). Tonotpic VGLUT1/2 expression did not differ based on genotype (*p* = 0.5658, 2-way ANOVA, *F* (2, 16) = 0.5903) (Figures 3A,B).

We previously found that loss of microglial fraktalkine receptor led to increased GLYT2 levels in the MNTB (Milinkeviciute et al., 2021a). Here, we tested whether C1q regulates inhibitory synapse levels in the superior olivary complex. We found that C1q KO did not alter GLYT2 expression in the MNTB (*p* > 0.9999, Mann–Whitney test) in the MNTB (Figures 3C,D). GLYT2 levels across the MNTB tonotopic axis were similar between WT and C1q KO mice (*p* = 0.5303, *F* (2, 16) = 0.6602, 2-way ANOVA) (Figure 3D). GLYT2 levels were also similar in the LSO (*p* > 0.9999, Mann–Whitney test) and did not differ based on tonotopic region (*p* = 0.5518, *F* (2, 14) = 0.6206, 2-way ANOVA) (Figure 3D). Together, these data show that congenital loss of C1q did not affect excitatory or inhibitory synapse levels in the superior olivary complex.

### C1q depletion alters the auditory brainstem responses

The ABR waveform reflects overall activity in the cochlea, spiral ganglion cells, and VIIIth nerve (peak I), cochlear nucleus (peak II), superior olivary complex which includes MNTB and LSO (peak III), and lateral lemniscus (peak IV) (Henry, 1979; Jewett, 1970; Jewett and Williston, 1971). Mice that underwent pharmacological microglia depletion showed significant hearing loss and abnormal auditory brainstem function, as detected by their elevated ABR thresholds, delayed peak latencies, and reduced peak amplitudes (Chokr et al., 2022; Milinkeviciute et al., 2021b). Some recovery in the ABR was detected following microglial return (Milinkeviciute et al., 2021b). Mice that lack the C1QL1 protein in the cochlea also have extensive hearing loss as shown by their elevated hearing thresholds and delayed peak 1 latencies (Qi et al., 2021). Here, we assessed whether loss of microglia-secreted C1q affects the mouse hearing profile. Seven wildtype (WT) and 11 C1q KO (KO) mice of both sexes at P28 were used for ABR comparisons. All ABR statistical analyses indicating peak amplitude, latency, and interpeak latency differences are shown in Supplementary Table S1. Statistical significance is reported below as *p* < 0.05.

**Figure 4:**
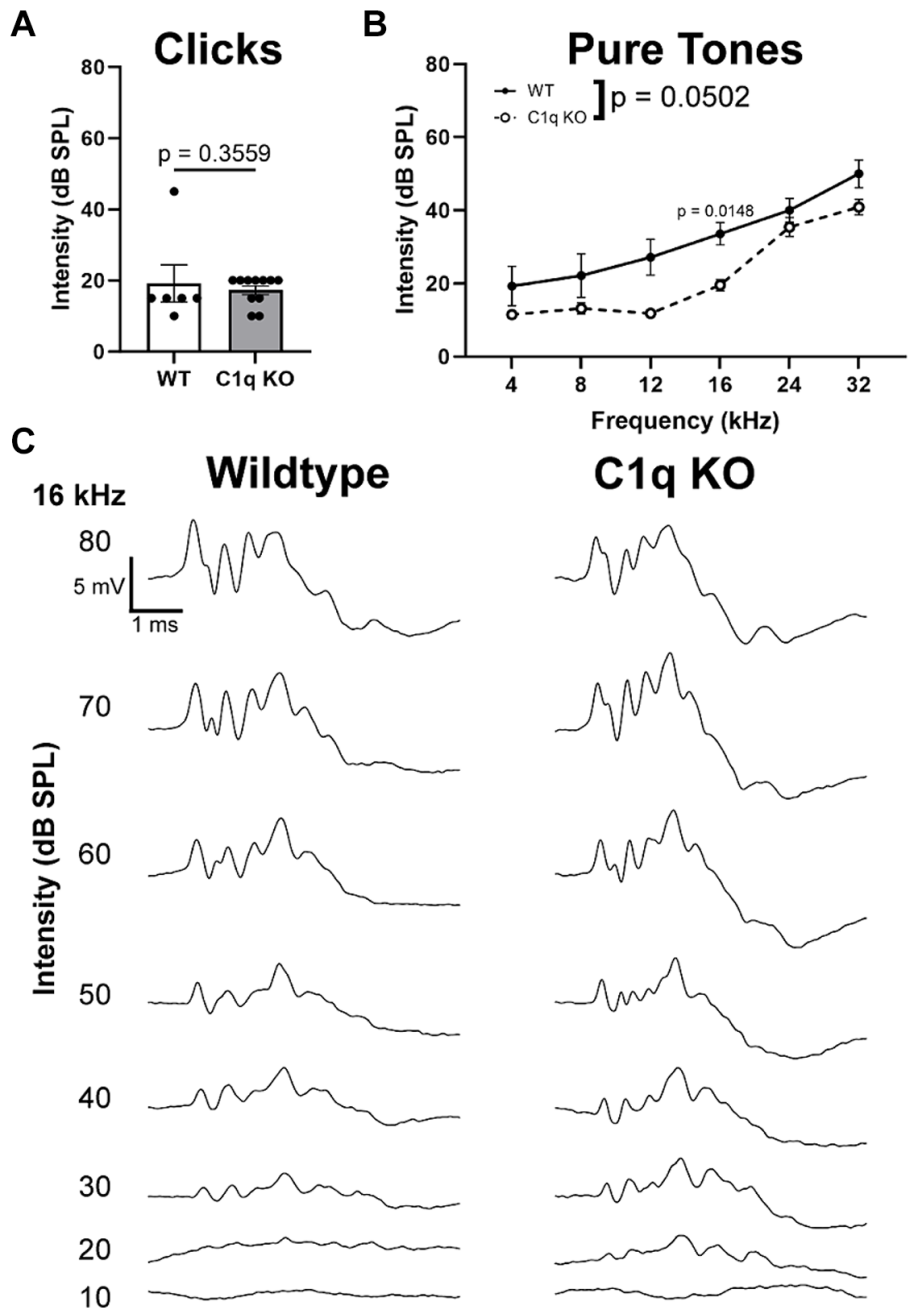
Hearing thresholds in C1q KO mice and controls. **(A)** Hearing thresholds did not differ between controls and C1q KO mice in response to click stimuli. **(B)** C1q KO mice had slightly decreased hearing thresholds compared to age-matched controls. **(C)** Sample traces for wildtype and C1q KO mice.

Click and pure tone (4, 8, 12, 16, 24, and 32 kHz) stimuli were presented to the left ear at decreasing intensities (80–10 dB SPL, 5 dB SPL increments). Hearing threshold in response to both click and pure tones and was defined as the lowest intensity at which peak I level (μV) was greater than or equal to 4 standard deviations from noise (Milinkeviciute et al., 2021b). Click thresholds in C1q KO mice were comparable to age-matched controls (*p* = 0.3559, Mann Whitney U = 24) (Figure 4A). C1q KO mice appeared to have significantly lower hearing thresholds at 16 kHz (*p* = 0.0148, *t* = 4.107, df = 9.272) (Figure 4B). All other tested frequencies were comparable to controls (4 kHz *p* = 0.7397, *t* = 1.429, df = 6.241; 8 kHz *p* = 0.7170, *t* = 6.818, df = 6.528; 12 kHz *p* = 0.1104, *t* = 3.085, df = 9.272, 24 kHz *p* = 0.8767, *t* = 1.093, df = 12.74; 32 kHz *p* = 0.3223, *t* = 2.100, df = 9.763) (Figure 4B). Sample traces in Figure 4C illustrate these values. These data show that C1q KO mouse hearing thresholds are largely comparable to age-matched controls with the exception of diminished thresholds at a central frequency.

**Figure 5:**
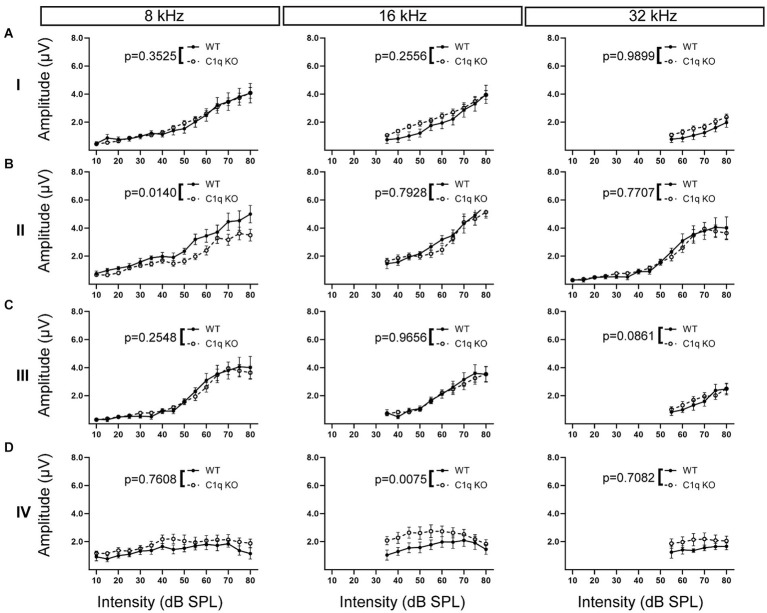
ABR peak amplitudes in C1q KO mice. **(A)** Peak I amplitudes in C1q KO mice were comparable to controls. **(B)** C1q KO mice had slightly diminished peak II amplitude at low frequencies but the mid to high frequencies were not altered. **(C)** Peak III amplitude was not altered from C1q deletion. **(D)** C1q KO peak IV amplitude was elevated in mid frequencies, but other frequencies were comparable to controls.

We tested the effects of C1q removal on ABR peak amplitudes. Peak amplitude was defined as the difference in level (μV) between the apex of the peak and its preceding trough. All statistical analyses are detailed in Supplementary Table S1. C1q KO mice showed normal peak I amplitudes at every frequency tested (Supplementary Table S1; Figure 5A). At peak II, C1q KO mice showed decreased peak amplitude at 8 kHz, but the other frequencies tested did not show any differences (Supplementary Table S1; Figure 5B). At the level of the SOC, peak III amplitudes in C1q KO were comparable to controls at every frequency level (Supplementary Table S1; Figure 5C). Peak IV amplitudes, which reflect the projections of SOC activity to the LL along the ascending auditory pathway, showed significantly elevated peak amplitudes at 4, 12, 16, and 24 kHz (Supplementary Table S1; Figure 5D). Taken together, C1q KO mice showed largely unaffected peak amplitudes in the earlier peaks until peak IV, where peak amplitudes appeared larger than those of age-matched controls. Increases in amplitude may reflect deficits in auditory processing (Abadi et al., 2016; Schaette and McAlpine, 2011).

**Figure 6:**
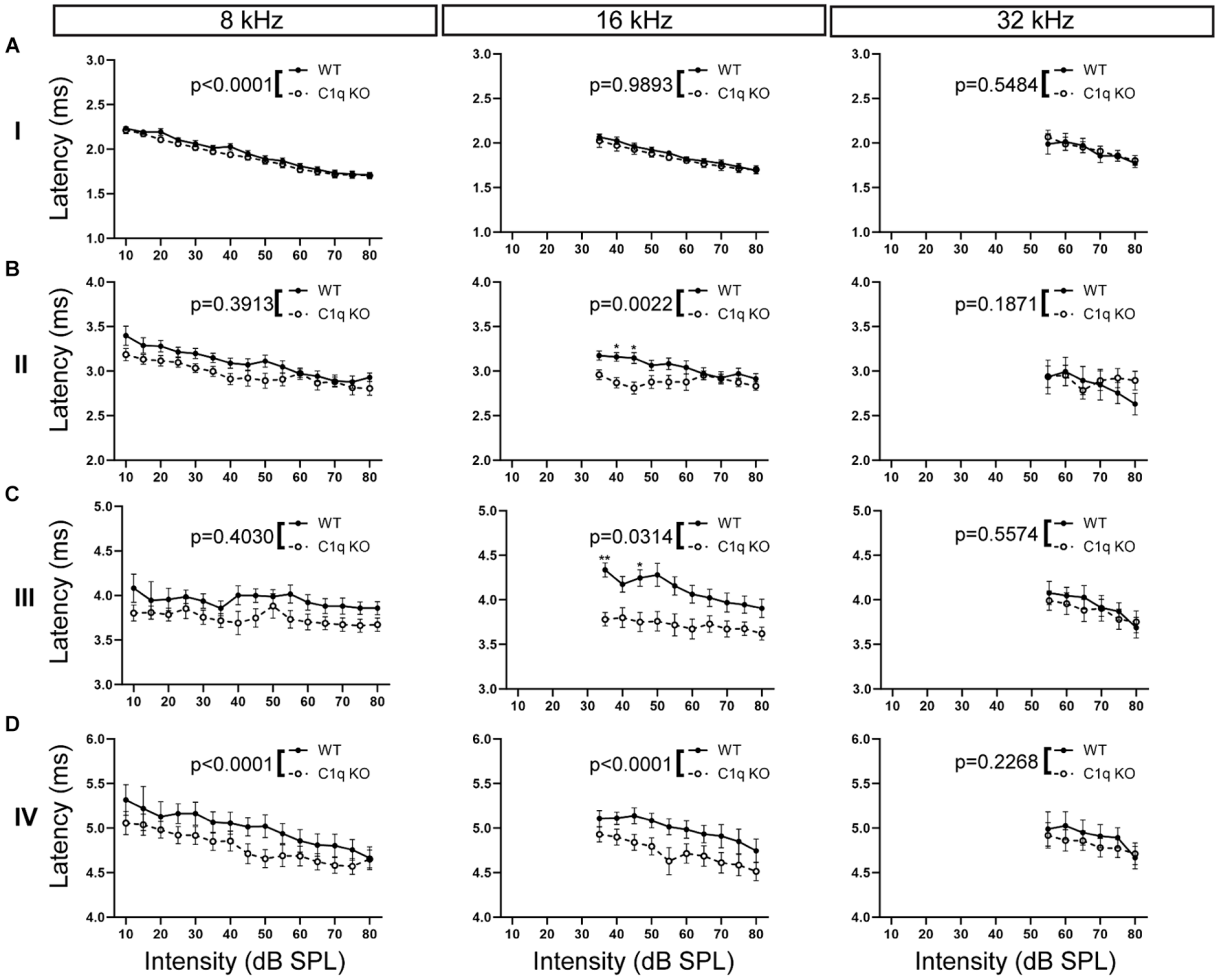
ABR peak latencies are decreased in C1q KO mice. **(A)** Peak I latencies were comparable in each frequency tested. **(B)** Peak II latency was shortened in the central frequencies in C1q KO mice. **(C)** Peak III latencies were diminished in mid frequencies in C1q KO mice. **(D)** C1q KO mice had shortened peak latencies at low and middle frequencies compared to age-matched controls.

Loss of microglia leads to delayed peak latencies, especially at the lower frequencies, whereas the elimination of microglial fractalkine signaling decreases peak latencies, especially at the higher frequencies (Chokr et al., 2022; Milinkeviciute et al., 2021a; Milinkeviciute et al., 2021b). Here, we assessed whether loss of C1q affects peak latencies along the ascending auditory pathway. C1q KO mice showed significantly decreased peak I latencies at 4, 8, and 12 kHz, the mid to high frequencies were not affected (Supplementary Table S1; Figure 6A). At peak II, C1q KO mice showed significantly reduced peak latencies at 12 and 16 kHz, the other frequencies were not affected (Supplementary Table S1; Figure 6B). Peak III latencies were significantly reduced at 4, 12, and 16 kHz in the C1q KO mice (Supplementary Table S1; Figure 6C). At peak IV, peak latencies in C1q KO were significantly reduced at 4 and 8 kHz, compared to age-matched controls (Supplementary Table S1; Figure 6D). Overall, it appears that peak latencies are shortened in C1q KO mice, particularly for the lower frequencies.

**Figure 7:**
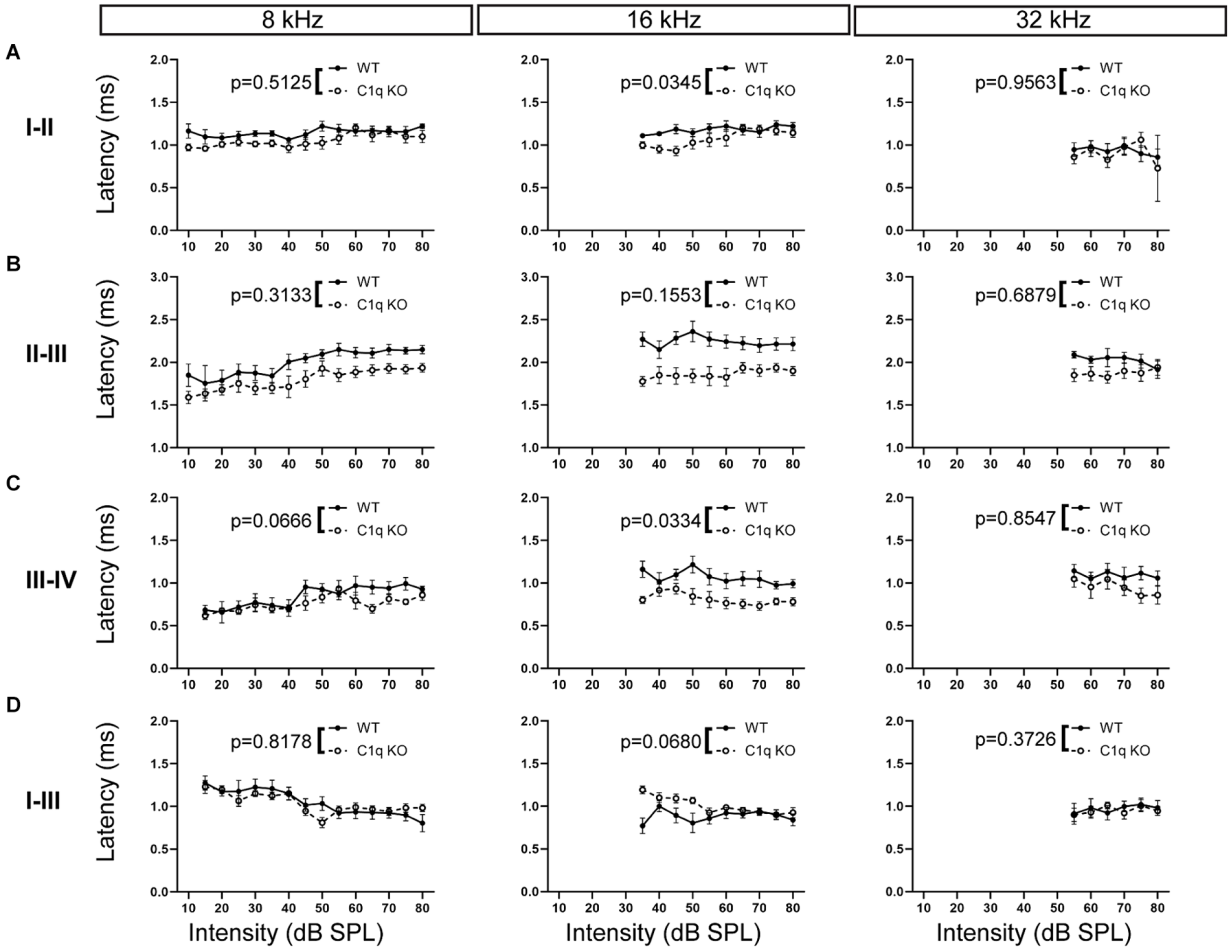
ABR interpeak latencies in C1q KO mice and age-matched controls. **(A)** C1q deletion diminished peak I–II interpeak latency at 16 kHz, while other frequencies were unaffected. **(B)** Peak II–III interpeak latencies were comparable to controls across all frequency levels. **(C)** Peak III–IV latency was diminished at 16 kHz. **(D)** Peak I–III interpeak latency was unaltered from C1q deletion.

We assessed central latency effects by measuring interpeak latencies in C1q KO and their age-matched wild type controls. Interpeak latency was defined as the difference in time (ms) between the apex of the peak and the apex of the preceding peak. C1q KO mice had significantly decreased peak I–II latency at 16 kHz, while the other frequencies tested were not affected (Supplementary Table S1; Figure 7A). Latencies between peaks II–III were unaffected in C1q KO mice at all frequencies (Supplementary Table S1; Figure 7B). Interpeak latency was shortened between peaks III–IV at 16 kHz, while the other frequencies remained unaffected by C1q depletion (Supplementary Table S1; Figure 7C). Next, we assessed overall interpeak latencies effects by examining latency differences between peaks I–III and I–IV. At 4 kHz, peak I–III interpeak latencies were significantly shortened in C1q KO mice (Supplementary Table S1; Figure 7D). Peak I–IV latencies were unaffected at all frequency levels (Supplementary Table S1). Together, the interpeak latencies appear disrupted at some low to mid frequency regions.

## Discussion

In this study we investigated the role of the classic complement cascade initiator, C1q, in the context of auditory brainstem development. We found that C1q is present throughout the brainstem before and after hearing onset. C1q expression increases by P14, a period of microglia-regulated circuit refinement. We observed that microglia contain C1q within their soma and processes, and C1q expression is more prominent surrounding MNTB cells after hearing onset. Further, C1q closely surrounds VGLUT2 puncta, indicating its calyceal interactions. Loss of C1q, however, does not affect monoinnervation in the MNTB. C1q ablation does not alter the E/I protein ratio in the auditory brainstem. C1q KO mice appeared to have mostly normal ABR hearing thresholds. However, we found that loss of C1q leads to decreased peak latencies, particularly in the lower frequencies tested.

### Dispersion of C1q protein in the developing brain

In C57/BL6 mice C1q levels increase during development, followed by a sharp increase in older animals (Reichwald et al., 2009; Rupprecht et al., 2021; Stephan et al., 2013). Throughout the brain, C1q expression rises between P6 to 15 and P15 to 30 (Stephan et al., 2013). In the developing lateral geniculate nucleus (LGN), it was found that C1q peaks in expression in the first two postnatal weeks, in contrast with C1q downregulation in adulthood (Stevens et al., 2007). C1q increases are predominantly found at the synapse and can influence synaptic plasticity outside of the classical complement cascade (Stephan et al., 2013; Stevens et al., 2007). Here, we found that C1q levels in the MNTB increase with age. We also found a gradient shift where at P8, earlier developing high frequency regions have higher C1q expression, and at P14 low frequency regions have higher C1q expression. Developing microglia contain C1q clusters within their soma and processes, and non-microglial C1q is more apparent at older ages. These findings imply that C1q follows a developmental sequence during auditory circuit optimization.

C1q roles are heterogeneous depending on brain region and age. Hippocampal development entails C1q and C3-mediated synaptic pruning (Paolicelli et al., 2011). C1q KO mice have deficiencies in retinogeniculate refinement, and LGN neurons remain polyinnervated with C1q removal (Stevens et al., 2007). During a period of robust activity-dependent pruning in the postnatal retinogeniculate system, microglia engulf retinal ganglion cells in a C3-dependent way (Schafer et al., 2012). C1q is not required for the development of a spine population implicated in ocular dominance plasticity in the binocular primary visual cortex (Welsh et al., 2020). Dendritic morphology is not dependent on C1q removal in this model system and firing rates of V1b in C1q KO mice are normal (Welsh et al., 2020). Using a monocular deprivation (MD) paradigm, C1q protein levels in the primary visual cortex were not experience-dependent, and MD did not reduce spine numbers on L2/3 pyramidal neurons in C1q knock-out mice (Welsh et al., 2020). In a model of demyelinating disease, C1q depletion rescues the number and function of synapses (Vukojicic et al., 2019). Here, we observed C1q expression surrounding MNTB cells and in close proximity to calyx-associated excitatory puncta. We did not find changes in calyx elimination following C1q removal or in our assessment of synaptic protein expression. Our findings further emphasize that C1q has heterogeneous roles in synapse elimination. It should be noted that C1q docking in calyceal regions may have synapse protection roles that should be investigated.

### C1q modulates auditory signal transmission

Microglia play a pivotal role in auditory brainstem formation and loss of microglia through CSF1R inhibition results in hearing impairments such as higher ABR thresholds and delayed peak latencies (Chokr et al., 2022; Milinkeviciute et al., 2021b). Interestingly, disruption of CX3CR1, a major microglial signaling pathway, results in shortened peak latencies (Milinkeviciute et al., 2021a).

Connections in the brainstem are highly myelinated, and disruptions in myelination can affect the ABR (Ito et al., 2004; Long et al., 2018; Sinclair et al., 2017; Xing et al., 2012). C1q may activate myelin by binding to myelin oligodendrocyte glycoprotein, potentially playing a role in demyelinating diseases (Johns and Bernard, 1997). Indeed, in a cuprizone-induced demyelination mouse model, C1q protein levels are elevated (Gao et al., 2022). C1q KO prevents white matter loss (Leah et al., 2020). C1q KO mice have increased spontaneous and evoked epileptiform activity, and increased excitatory connectivity, which can be detected through frequent behavioral seizures (Chu et al., 2010). C1q inhibition is a potent therapeutic target for demyelinating diseases and conditions with disrupted E/I balance such as epilepsy or schizophrenia (Dalakas et al., 2020; Lansita et al., 2017; Maes et al., 2021; McGonigal et al., 2016). Our ABR results show elevated peak amplitudes and decreased peak latencies, which are consistent with phenotypes that model increased myelination and a disrupted E/I balance. We postulate that although elevated peak amplitudes and decreased peak latencies can be a sign of an improved ABR profile, it is possible that these changes may indicate hyperactivity and excitability in the ascending auditory pathway. Detailed electrophysiological studies that investigate the effects of microglial signaling pathways on myelination in the sound localization pathway are currently lacking. These findings unravel a new avenue to explore the interplay of C1q and myelin in the context of auditory brainstem development and function.

## Conclusion

We showed here that C1q is present in the developing auditory brainstem. C1q protein is predominantly within microglia prior to hearing onset and increases in expression and surrounds MNTB cells after hearing onset. C1q molecules also surround calyceal excitatory puncta but loss of C1q does not affect calyceal pruning or the E/I synaptic ratio in the MNTB or LSO. Auditory brainstem responses revealed that loss of C1q shortens signal transmission, a phenotype suggesting altered myelination or neural strength.

## Data Availability

The original contributions presented in the study are included in the article/Supplementary material, further inquiries can be directed to the corresponding author.
