# Supplementary material for: Loss of C1q alters the auditory brainstem response
Source: Front Cell Neurosci. 2024 Oct 2;18:1464670. doi: 10.3389/fncel.2024.1464670 (PMC11480778; doi:10.3389/fncel.2024.1464670)
Supplement: Supplementary file 1 [file Table_1.docx]

| **Supplementary Table 1. ABR analyses** | |  |  |  |  |  |
| --- | --- | --- | --- | --- | --- | --- |
| **Mixed effects analysis** | |  |  |  |  |  |
| **Latencies** |  |  |  |  | **Amplitudes** |  |
| **Peak** | **Frequency (kHz)** | **Fixed effects (type III)** | **P value** | **F (DFn, DFd)** | **P value** | **F (DFn, DFd)** |
| **I** | **4** | Intensity | <0.0001 | F (2.870, 41.40) = 339.4 | <0.0001 | F (1.440, 19.65) = 147.3 |
|  |  | Genotype | 0.4528 | F (1, 16) = 0.5922 | 0.5978 | F (1, 15) = 0.2905 |
|  |  | Intensity x Genotype | 0.0025 | F (14, 202) = 2.517 | 0.1813 | F (14, 191) = 1.351 |
|  | **8** | Intensity | <0.0001 | F (3.166, 45.23) = 334.0 | <0.0001 | F (1.383, 19.66) = 119.6 |
|  |  | Genotype | 0.3075 | F (1, 16) = 1.111 | 0.5552 | F (1, 16) = 0.3632 |
|  |  | Intensity x Genotype | <0.0001 | F (14, 200) = 3.445 | 0.3525 | F (14, 199) = 1.108 |
|  | **12** | Intensity | <0.0001 | F (2.589, 36.84) = 171.4 | <0.0001 | F (1.476, 22.28) = 91.91 |
|  |  | Genotype | 0.2388 | F (1, 16) = 1.498 | 0.3223 | F (1, 16) = 1.043 |
|  |  | Intensity x Genotype | <0.0001 | F (13, 185) = 5.805 | 0.9969 | F (11, 166) = 0.2091 |
|  | **16** | Intensity | <0.0001 | F (1.235, 19.35) = 36.96 | <0.0001 | F (1.276, 19.99) = 89.56 |
|  |  | Genotype | 0.6011 | F (1, 16) = 0.2846 | 0.2972 | F (1, 16) = 1.161 |
|  |  | Intensity x Genotype | 0.9893 | F (9, 141) = 0.2323 | 0.2556 | F (9, 141) = 1.275 |
|  | **24** | Intensity | <0.0001 | F (2.786, 42.18) = 17.11 | <0.0001 | F (1.987, 30.37) = 93.70 |
|  |  | Genotype | 0.9586 | F (1, 16) = 0.002785 | 0.1087 | F (1, 16) = 2.887 |
|  |  | Intensity x Genotype | 0.696 | F (7, 106) = 0.6711 | 0.9369 | F (7, 107) = 0.3338 |
|  | **32** | Intensity | <0.0001 | F (2.435, 35.55) = 32.23 | <0.0001 | F (2.241, 34.96) = 55.27 |
|  |  | Genotype | 0.9185 | F (1, 15) = 0.01082 | 0.1614 | F (1, 16) = 2.156 |
|  |  | Intensity x Genotype | 0.5484 | F (5, 73) = 0.8070 | 0.9889 | F (5, 78) = 0.1143 |
| **II** | **4** | Intensity | <0.0001 | F (3.065, 41.60) = 9.619 | 0.0005 | F (1.571, 21.44) = 12.82 |
|  |  | Genotype | 0.1975 | F (1, 15) = 1.819 | 0.238 | F (1, 15) = 1.510 |
|  |  | Intensity x Genotype | 0.0902 | F (14, 190) = 1.572 | 0.8302 | F (14, 191) = 0.6390 |
|  | **8** | Intensity | <0.0001 | F (3.911, 55.59) = 17.91 | <0.0001 | F (1.927, 27.38) = 40.56 |
|  |  | Genotype | 0.1573 | F (1, 16) = 2.202 | 0.1505 | F (1, 16) = 2.281 |
|  |  | Intensity x Genotype | 0.3913 | F (14, 199) = 1.065 | 0.014 | F (14, 199) = 2.086 |
|  | **12** | Intensity | 0.0034 | F (3.107, 46.89) = 5.139 | <0.0001 | F (2.512, 37.91) = 38.32 |
|  |  | Genotype | 0.0499 | F (1, 16) = 4.496 | 0.4412 | F (1, 16) = 0.6237 |
|  |  | Intensity x Genotype | 0.0193 | F (11, 166) = 2.154 | 0.6813 | F (11, 166) = 0.7580 |
|  | **16** | Intensity | 0.1092 | F (1.843, 28.87) = 2.434 | <0.0001 | F (1.982, 31.04) = 42.09 |
|  |  | Genotype | 0.0435 | F (1, 16) = 4.806 | 0.6429 | F (1, 16) = 0.2233 |
|  |  | Intensity x Genotype | 0.0022 | F (9, 141) = 3.065 | 0.7928 | F (9, 141) = 0.6031 |
|  | **24** | Intensity | 0.0045 | F (3.232, 48.94) = 4.772 | 0.0006 | F (2.364, 35.79) = 8.268 |
|  |  | Genotype | 0.8947 | F (1, 16) = 0.01810 | 0.4835 | F (1, 16) = 0.5145 |
|  |  | Intensity x Genotype | 0.1186 | F (7, 106) = 1.692 | 0.7485 | F (7, 106) = 0.6077 |
|  | **32** | Intensity | 0.2042 | F (2.382, 36.68) = 1.639 | 0.689 | F (2.539, 38.08) = 0.4470 |
|  |  | Genotype | 0.7454 | F (1, 16) = 0.1091 | 0.7993 | F (1, 16) = 0.06686 |
|  |  | Intensity x Genotype | 0.1871 | F (5, 77) = 1.541 | 0.7707 | F (5, 75) = 0.5061 |
| **III** | **4** | Intensity | 0.016 | F (3.131, 39.59) = 3.810 | <0.0001 | F (2.111, 27.14) = 27.75 |
|  |  | Genotype | 0.0698 | F (1, 15) = 3.812 | 0.7412 | F (1, 15) = 0.1132 |
|  |  | Intensity x Genotype | 0.004 | F (14, 177) = 2.421 | 0.2897 | F (14, 180) = 1.186 |
|  | **8** | Intensity | 0.0025 | F (3.011, 42.36) = 5.588 | <0.0001 | F (1.432, 20.04) = 61.99 |
|  |  | Genotype | 0.0966 | F (1, 16) = 3.116 | 0.6374 | F (1, 16) = 0.2309 |
|  |  | Intensity x Genotype | 0.403 | F (14, 197) = 1.053 | 0.2548 | F (14, 196) = 1.232 |
|  | **12** | Intensity | <0.0001 | F (2.794, 41.15) = 12.90 | <0.0001 | F (1.588, 23.68) = 50.07 |
|  |  | Genotype | 0.0331 | F (1, 16) = 5.436 | 0.4738 | F (1, 16) = 0.5382 |
|  |  | Intensity x Genotype | 0.0052 | F (11, 162) = 2.560 | 0.9177 | F (11, 164) = 0.4732 |
|  | **16** | Intensity | 0.0048 | F (1.301, 20.38) = 8.717 | <0.0001 | F (1.494, 23.40) = 39.01 |
|  |  | Genotype | 0.0095 | F (1, 16) = 8.664 | 0.8765 | F (1, 16) = 0.02495 |
|  |  | Intensity x Genotype | 0.0134 | F (9, 141) = 2.431 | 0.9656 | F (9, 141) = 0.3251 |
|  | **24** | Intensity | 0.0112 | F (1.706, 25.84) = 5.767 | <0.0001 | F (2.552, 38.64) = 16.36 |
|  |  | Genotype | 0.188 | F (1, 16) = 1.892 | 0.1642 | F (1, 16) = 2.126 |
|  |  | Intensity x Genotype | 0.7639 | F (7, 106) = 0.5886 | 0.907 | F (7, 106) = 0.3890 |
|  | **32** | Intensity | 0.0017 | F (2.302, 35.45) = 7.101 | <0.0001 | F (2.696, 41.52) = 29.06 |
|  |  | Genotype | 0.7254 | F (1, 16) = 0.1278 | 0.6655 | F (1, 16) = 0.1941 |
|  |  | Intensity x Genotype | 0.5574 | F (5, 77) = 0.7939 | 0.0861 | F (5, 77) = 2.013 |
| **IV** | **4** | Intensity | <0.0001 | F (2.474, 30.93) = 16.15 | 0.0007 | F (3.891, 48.64) = 5.882 |
|  |  | Genotype | 0.1658 | F (1, 15) = 2.122 | 0.2153 | F (1, 15) = 1.674 |
|  |  | Intensity x Genotype | 0.0099 | F (14, 175) = 2.187 | 0.0362 | F (14, 175) = 1.840 |
|  | **8** | Intensity | <0.0001 | F (3.444, 47.73) = 25.32 | <0.0001 | F (3.596, 49.83) = 11.16 |
|  |  | Genotype | 0.1581 | F (1, 16) = 2.192 | 0.2375 | F (1, 16) = 1.506 |
|  |  | Intensity x Genotype | 0.0478 | F (14, 194) = 1.756 | 0.7608 | F (14, 194) = 0.7121 |
|  | **12** | Intensity | <0.0001 | F (2.021, 29.40) = 21.30 | 0.0008 | F (3.688, 54.31) = 5.785 |
|  |  | Genotype | 0.0921 | F (1, 16) = 3.210 | 0.2351 | F (1, 16) = 1.522 |
|  |  | Intensity x Genotype | 0.7078 | F (11, 160) = 0.7308 | 0.0034 | F (11, 162) = 2.687 |
|  | **16** | Intensity | 0.0005 | F (1.328, 20.51) = 14.22 | 0.0117 | F (1.175, 18.15) = 7.273 |
|  |  | Genotype | 0.0792 | F (1, 16) = 3.516 | 0.1653 | F (1, 16) = 2.114 |
|  |  | Intensity x Genotype | 0.8558 | F (9, 139) = 0.5233 | 0.0075 | F (9, 139) = 2.640 |
|  | **24** | Intensity | 0.0006 | F (1.656, 24.37) = 11.53 | 0.1268 | F (2.098, 31.16) = 2.190 |
|  |  | Genotype | 0.0793 | F (1, 16) = 3.512 | 0.2621 | F (1, 16) = 1.351 |
|  |  | Intensity x Genotype | 0.6738 | F (7, 103) = 0.6978 | 0.0391 | F (7, 104) = 2.211 |
|  | **32** | Intensity | 0.0063 | F (2.211, 34.05) = 5.628 | 0.4874 | F (2.835, 43.09) = 0.8137 |
|  |  | Genotype | 0.5944 | F (1, 16) = 0.2951 | 0.2951 | F (1, 16) = 1.172 |
|  |  | Intensity x Genotype | 0.5117 | F (5, 77) = 0.8604 | 0.7082 | F (5, 76) = 0.5892 |
| **Inter-peak latency** | |  |  |  |  |  |
| **Peak** | **Frequency (kHz)** | Fixed effects (type III) | P value | F (DFn, DFd) |  |  |
| **I-II** | **4** | Intensity | 0.029 | F (3.003, 40.75) = 3.322 |  |  |
|  |  | Genotype | 0.2585 | F (1, 15) = 1.380 |  |  |
|  |  | Intensity x Genotype | 0.4566 | F (14, 190) = 0.9984 |  |  |
|  | **8** | Intensity | 0.0218 | F (4.223, 60.03) = 3.044 |  |  |
|  |  | Genotype | 0.262 | F (1, 16) = 1.352 |  |  |
|  |  | Intensity x Genotype | 0.5125 | F (14, 199) = 0.9441 |  |  |
|  | **12** | Intensity | 0.004 | F (2.682, 40.48) = 5.484 |  |  |
|  |  | Genotype | 0.0742 | F (1, 16) = 3.650 |  |  |
|  |  | Intensity x Genotype | 0.4213 | F (11, 166) = 1.031 |  |  |
|  | **16** | Intensity | 0.0323 | F (1.902, 29.79) = 3.935 |  |  |
|  |  | Genotype | 0.1244 | F (1, 16) = 2.629 |  |  |
|  |  | Intensity x Genotype | 0.0345 | F (9, 141) = 2.086 |  |  |
|  | **24** | Intensity | 0.0036 | F (3.293, 49.86) = 4.900 |  |  |
|  |  | Genotype | 0.9048 | F (1, 16) = 0.01477 |  |  |
|  |  | Intensity x Genotype | 0.2467 | F (7, 106) = 1.323 |  |  |
|  | **32** | Intensity | 0.5651 | F (1.330, 20.75) = 0.4494 |  |  |
|  |  | Genotype | 0.7928 | F (1, 16) = 0.07136 |  |  |
|  |  | Intensity x Genotype | 0.9563 | F (5, 78) = 0.2123 |  |  |
| **II-III** | **4** | Intensity | 0.2066 | F (3.871, 48.94) = 1.540 |  |  |
|  |  | Genotype | 0.041 | F (1, 15) = 5.001 |  |  |
|  |  | Intensity x Genotype | 0.7626 | F (14, 177) = 0.7100 |  |  |
|  | **8** | Intensity | 0.0158 | F (5.105, 67.15) = 3.008 |  |  |
|  |  | Genotype | 0.1278 | F (1, 15) = 2.598 |  |  |
|  |  | Intensity x Genotype | 0.3313 | F (13, 171) = 1.137 |  |  |
|  | **12** | Intensity | 0.0694 | F (3.996, 54.86) = 2.309 |  |  |
|  |  | Genotype | 0.0215 | F (1, 15) = 6.586 |  |  |
|  |  | Intensity x Genotype | 0.6343 | F (11, 151) = 0.8057 |  |  |
|  | **16** | Intensity | 0.0979 | F (1.953, 28.65) = 2.537 |  |  |
|  |  | Genotype | 0.0056 | F (1, 15) = 10.46 |  |  |
|  |  | Intensity x Genotype | 0.1553 | F (9, 132) = 1.497 |  |  |
|  | **24** | Intensity | 0.0017 | F (3.302, 46.70) = 5.634 |  |  |
|  |  | Genotype | 0.1507 | F (1, 15) = 2.293 |  |  |
|  |  | Intensity x Genotype | 0.2085 | F (7, 99) = 1.413 |  |  |
|  | **32** | Intensity | 0.445 | F (3.093, 44.55) = 0.9125 |  |  |
|  |  | Genotype | 0.2467 | F (1, 15) = 1.453 |  |  |
|  |  | Intensity x Genotype | 0.6879 | F (5, 72) = 0.6160 |  |  |
| **III-IV** | **4** | Intensity | 0.0024 | F (5.231, 65.39) = 4.085 |  |  |
|  |  | Genotype | 0.3149 | F (1, 15) = 1.081 |  |  |
|  |  | Intensity x Genotype | 0.698 | F (14, 175) = 0.7723 |  |  |
|  | **8** | Intensity | <0.0001 | F (4.587, 62.11) = 8.643 |  |  |
|  |  | Genotype | 0.9909 | F (1, 15) = 0.0001339 |  |  |
|  |  | Intensity x Genotype | 0.0666 | F (13, 176) = 1.689 |  |  |
|  | **12** | Intensity | 0.0003 | F (4.026, 54.54) = 6.206 |  |  |
|  |  | Genotype | 0.3678 | F (1, 15) = 0.8622 |  |  |
|  |  | Intensity x Genotype | 0.9797 | F (11, 149) = 0.3234 |  |  |
|  | **16** | Intensity | 0.0952 | F (3.892, 56.22) = 2.095 |  |  |
|  |  | Genotype | 0.0185 | F (1, 15) = 6.971 |  |  |
|  |  | Intensity x Genotype | 0.0034 | F (9, 130) = 2.930 |  |  |
|  | **24** | Intensity | 0.2704 | F (2.641, 36.22) = 1.362 |  |  |
|  |  | Genotype | 0.177 | F (1, 15) = 2.007 |  |  |
|  |  | Intensity x Genotype | 0.6679 | F (7, 96) = 0.7048 |  |  |
|  | **32** | Intensity | 0.7344 | F (3.091, 44.51) = 0.4355 |  |  |
|  |  | Genotype | 0.7685 | F (1, 15) = 0.08980 |  |  |
|  |  | Intensity x Genotype | 0.8547 | F (5, 72) = 0.3892 |  |  |
| **I-III** | **4** | Intensity | 0.0027 | F (3.491, 44.14) = 5.113 |  |  |
|  |  | Genotype | 0.0496 | F (1, 15) = 4.561 |  |  |
|  |  | Intensity x Genotype | 0.0192 | F (14, 177) = 2.012 |  |  |
|  | **8** | Intensity | <0.0001 | F (3.175, 42.18) = 10.41 |  |  |
|  |  | Genotype | 0.0334 | F (1, 15) = 5.484 |  |  |
|  |  | Intensity x Genotype | 0.8178 | F (14, 186) = 0.6526 |  |  |
|  | **12** | Intensity | <0.0001 | F (26, 248) = 2.776 |  |  |
|  |  | Genotype | <0.0001 | F (1, 248) = 100.1 |  |  |
|  |  | Intensity x Genotype |  |  |  |  |
|  | **16** | Intensity | 0.4513 | F (1.476, 21.65) = 0.7363 |  |  |
|  |  | Genotype | 0.0021 | F (1, 15) = 13.80 |  |  |
|  |  | Intensity x Genotype | 0.068 | F (9, 132) = 1.833 |  |  |
|  | **24** | Intensity | 0.4763 | F (2.018, 28.53) = 0.7637 |  |  |
|  |  | Genotype | 0.0991 | F (1, 15) = 3.092 |  |  |
|  |  | Intensity x Genotype | 0.9022 | F (7, 99) = 0.3968 |  |  |
|  | **32** | Intensity | 0.8781 | F (2.851, 41.05) = 0.2130 |  |  |
|  |  | Genotype | 0.1651 | F (1, 15) = 2.130 |  |  |
|  |  | Intensity x Genotype | 0.3726 | F (5, 72) = 1.091 |  |  |
| **I-IV** | **4** | Intensity | 0.2595 | F (1.203, 15.04) = 1.418 |  |  |
|  |  | Genotype | 0.5527 | F (1, 15) = 0.3688 |  |  |
|  |  | Intensity x Genotype | 0.078 | F (14, 175) = 1.619 |  |  |
|  | **8** | Intensity | 0.6106 | F (2.034, 26.87) = 0.5076 |  |  |
|  |  | Genotype | 0.0581 | F (1, 15) = 4.207 |  |  |
|  |  | Intensity x Genotype | 0.9866 | F (14, 185) = 0.3468 |  |  |
|  | **12** | Intensity | 0.2018 | F (2.687, 36.40) = 1.635 |  |  |
|  |  | Genotype | 0.0623 | F (1, 15) = 4.058 |  |  |
|  |  | Intensity x Genotype | 0.5099 | F (11, 149) = 0.9337 |  |  |
|  | **16** | Intensity | 0.485 | F (1.453, 21.15) = 0.6494 |  |  |
|  |  | Genotype | 0.0725 | F (1, 15) = 3.731 |  |  |
|  |  | Intensity x Genotype | 0.591 | F (9, 131) = 0.8286 |  |  |
|  | **24** | Intensity | 0.4019 | F (2.626, 36.01) = 0.9854 |  |  |
|  |  | Genotype | 0.0363 | F (1, 15) = 5.283 |  |  |
|  |  | Intensity x Genotype | 0.3801 | F (7, 96) = 1.083 |  |  |
|  | **32** | Intensity | 0.3567 | F (2.695, 38.81) = 1.100 |  |  |
|  |  | Genotype | 0.1315 | F (1, 15) = 2.545 |  |  |
|  |  | Intensity x Genotype | 0.2146 | F (5, 72) = 1.457 |  |  |
| **Multiple comparisons analysis** | | |  |  |  |  |
| **Latencies** |  |  |  |  |  |  |
| **Peak** | **Frequency (kHz)** | **Intensity (dB SPL)** | **WT Mean (mV)** | **C1q KO Mean (mV)** | **P value** |  |
| **1** | **4** | 10 | 2.23 | 2.189 | >0.9999 |  |
|  |  | 15 | 2.153 | 2.162 | >0.9999 |  |
|  |  | 20 | 2.063 | 2.072 | >0.9999 |  |
|  |  | 25 | 2.006 | 2.021 | >0.9999 |  |
|  |  | 30 | 1.965 | 1.98 | >0.9999 |  |
|  |  | 35 | 1.924 | 1.954 | >0.9999 |  |
|  |  | 40 | 2.002 | 1.917 | 0.9303 |  |
|  |  | 45 | 1.949 | 1.879 | 0.9755 |  |
|  |  | 50 | 1.908 | 1.85 | 0.9852 |  |
|  |  | 55 | 1.867 | 1.812 | 0.9962 |  |
|  |  | 60 | 1.826 | 1.782 | 0.9992 |  |
|  |  | 65 | 1.773 | 1.756 | >0.9999 |  |
|  |  | 70 | 1.732 | 1.734 | >0.9999 |  |
|  |  | 75 | 1.714 | 1.7 | >0.9999 |  |
|  |  | 80 | 1.679 | 1.689 | >0.9999 |  |
|  | **8** | 10 | 2.233 | 2.213 | >0.9999 |  |
|  |  | 15 | 2.192 | 2.174 | >0.9999 |  |
|  |  | 20 | 2.195 | 2.107 | 0.7876 |  |
|  |  | 25 | 2.104 | 2.063 | 0.9935 |  |
|  |  | 30 | 2.063 | 2.018 | 0.9983 |  |
|  |  | 35 | 2.014 | 1.973 | 0.9985 |  |
|  |  | 40 | 2.028 | 1.939 | 0.696 |  |
|  |  | 45 | 1.95 | 1.909 | 0.9997 |  |
|  |  | 50 | 1.891 | 1.868 | >0.9999 |  |
|  |  | 55 | 1.867 | 1.831 | >0.9999 |  |
|  |  | 60 | 1.809 | 1.771 | 0.9997 |  |
|  |  | 65 | 1.773 | 1.749 | >0.9999 |  |
|  |  | 70 | 1.732 | 1.715 | >0.9999 |  |
|  |  | 75 | 1.72 | 1.711 | >0.9999 |  |
|  |  | 80 | 1.708 | 1.704 | >0.9999 |  |
|  | **12** | 15 | 2.22 | 2.129 | 0.9998 |  |
|  |  | 20 | 2.137 | 2.059 | >0.9999 |  |
|  |  | 25 | 2.082 | 2.006 | 0.9325 |  |
|  |  | 30 | 2.028 | 1.98 | 0.9984 |  |
|  |  | 35 | 2.055 | 1.939 | 0.7169 |  |
|  |  | 40 | 1.991 | 1.902 | 0.5257 |  |
|  |  | 45 | 1.932 | 1.876 | 0.8991 |  |
|  |  | 50 | 1.891 | 1.831 | 0.9102 |  |
|  |  | 55 | 1.838 | 1.793 | 0.9991 |  |
|  |  | 60 | 1.803 | 1.763 | 0.9998 |  |
|  |  | 65 | 1.75 | 1.737 | >0.9999 |  |
|  |  | 70 | 1.714 | 1.711 | >0.9999 |  |
|  |  | 75 | 1.691 | 1.7 | >0.9999 |  |
|  |  | 80 | 1.685 | 1.723 | 0.9997 |  |
|  | **16** | 35 | 2.065 | 2.025 | >0.9999 |  |
|  |  | 40 | 2.026 | 1.973 | 0.9989 |  |
|  |  | 45 | 1.961 | 1.928 | >0.9999 |  |
|  |  | 50 | 1.92 | 1.879 | 0.9964 |  |
|  |  | 55 | 1.885 | 1.838 | 0.9524 |  |
|  |  | 60 | 1.82 | 1.805 | >0.9999 |  |
|  |  | 65 | 1.797 | 1.764 | 0.9991 |  |
|  |  | 70 | 1.773 | 1.741 | >0.9999 |  |
|  |  | 75 | 1.732 | 1.711 | >0.9999 |  |
|  |  | 80 | 1.691 | 1.7 | >0.9999 |  |
|  | **24** | 45 | 2.162 | 2.22 | >0.9999 |  |
|  |  | 50 | 2.067 | 1.932 | 0.909 |  |
|  |  | 55 | 1.873 | 1.865 | >0.9999 |  |
|  |  | 60 | 1.82 | 1.82 | >0.9999 |  |
|  |  | 65 | 1.796 | 1.784 | >0.9999 |  |
|  |  | 70 | 1.773 | 1.797 | >0.9999 |  |
|  |  | 75 | 1.779 | 1.764 | >0.9999 |  |
|  |  | 80 | 1.738 | 1.752 | >0.9999 |  |
|  | **32** | 55 | 1.989 | 2.068 | 0.9949 |  |
|  |  | 60 | 2.014 | 1.989 | >0.9999 |  |
|  |  | 65 | 1.973 | 1.952 | >0.9999 |  |
|  |  | 70 | 1.855 | 1.907 | 0.9955 |  |
|  |  | 75 | 1.855 | 1.854 | >0.9999 |  |
|  |  | 80 | 1.773 | 1.804 | 0.999 |  |
| **2** | **4** | 10 | 3.319 | 3.196 | 0.9971 |  |
|  |  | 15 | 3.222 | 3.119 | 0.988 |  |
|  |  | 20 | 3.132 | 3.009 | 0.9436 |  |
|  |  | 25 | 3.074 | 3.009 | 0.9998 |  |
|  |  | 30 | 3.042 | 2.984 | >0.9999 |  |
|  |  | 35 | 3.017 | 2.935 | 0.9995 |  |
|  |  | 40 | 3.1 | 2.885 | 0.7497 |  |
|  |  | 45 | 3.124 | 2.795 | 0.1181 |  |
|  |  | 50 | 3.03 | 2.75 | 0.54 |  |
|  |  | 55 | 2.965 | 2.803 | 0.9954 |  |
|  |  | 60 | 3.03 | 2.963 | >0.9999 |  |
|  |  | 65 | 2.987 | 2.91 | >0.9999 |  |
|  |  | 70 | 2.895 | 2.857 | >0.9999 |  |
|  |  | 75 | 2.853 | 2.832 | >0.9999 |  |
|  |  | 80 | 2.848 | 2.815 | >0.9999 |  |
|  | **8** | 10 | 3.398 | 3.185 | 0.9441 |  |
|  |  | 15 | 3.288 | 3.133 | 0.9674 |  |
|  |  | 20 | 3.28 | 3.115 | 0.7417 |  |
|  |  | 25 | 3.214 | 3.097 | 0.9182 |  |
|  |  | 30 | 3.198 | 3.034 | 0.5443 |  |
|  |  | 35 | 3.148 | 2.997 | 0.6971 |  |
|  |  | 40 | 3.089 | 2.911 | 0.5662 |  |
|  |  | 45 | 3.071 | 2.922 | 0.9651 |  |
|  |  | 50 | 3.112 | 2.892 | 0.591 |  |
|  |  | 55 | 3.047 | 2.907 | 0.9468 |  |
|  |  | 60 | 2.971 | 2.974 | >0.9999 |  |
|  |  | 65 | 2.942 | 2.866 | 0.9999 |  |
|  |  | 70 | 2.889 | 2.881 | >0.9999 |  |
|  |  | 75 | 2.877 | 2.814 | >0.9999 |  |
|  |  | 80 | 2.93 | 2.806 | 0.9614 |  |
|  | **12** | 25 | 3.165 | 2.974 | 0.6899 |  |
|  |  | 30 | 3.096 | 2.952 | 0.6634 |  |
|  |  | 35 | 3.124 | 2.941 | 0.7538 |  |
|  |  | 40 | 3.229 | 2.899 | 0.1975 |  |
|  |  | 45 | 3.071 | 2.87 | 0.4416 |  |
|  |  | 50 | 3.083 | 2.885 | 0.5325 |  |
|  |  | 55 | 3.042 | 2.997 | >0.9999 |  |
|  |  | 60 | 3.053 | 2.941 | 0.931 |  |
|  |  | 65 | 3.001 | 2.896 | 0.9752 |  |
|  |  | 70 | 2.971 | 2.877 | 0.9725 |  |
|  |  | 75 | 2.93 | 2.851 | 0.9938 |  |
|  |  | 80 | 2.924 | 2.84 | 0.98 |  |
|  | **16** | 35 | 3.175 | 2.959 | 0.1595 |  |
|  |  | 40 | 3.159 | 2.87 | 0.0136 |  |
|  |  | 45 | 3.147 | 2.81 | 0.0181 |  |
|  |  | 50 | 3.065 | 2.877 | 0.4438 |  |
|  |  | 55 | 3.083 | 2.877 | 0.4322 |  |
|  |  | 60 | 3.041 | 2.877 | 0.8705 |  |
|  |  | 65 | 2.971 | 2.952 | >0.9999 |  |
|  |  | 70 | 2.924 | 2.918 | >0.9999 |  |
|  |  | 75 | 2.971 | 2.873 | 0.9302 |  |
|  |  | 80 | 2.912 | 2.832 | 0.9752 |  |
|  | **24** | 45 | 2.99 | 3.165 | 0.9976 |  |
|  |  | 50 | 3.1 | 2.795 | 0.7967 |  |
|  |  | 55 | 2.736 | 2.634 | 0.9948 |  |
|  |  | 60 | 2.671 | 2.541 | 0.9569 |  |
|  |  | 65 | 2.777 | 2.754 | >0.9999 |  |
|  |  | 70 | 2.777 | 2.892 | 0.9967 |  |
|  |  | 75 | 2.789 | 2.892 | 0.9993 |  |
|  |  | 80 | 2.783 | 2.825 | >0.9999 |  |
|  | **32** | 55 | 2.935 | 2.937 | >0.9999 |  |
|  |  | 60 | 2.995 | 2.952 | >0.9999 |  |
|  |  | 65 | 2.895 | 2.784 | 0.9932 |  |
|  |  | 70 | 2.848 | 2.892 | >0.9999 |  |
|  |  | 75 | 2.754 | 2.922 | 0.8911 |  |
|  |  | 80 | 2.63 | 2.894 | 0.5495 |  |
| **3** | **4** | 10 | 4.059 | 3.792 | 0.9478 |  |
|  |  | 15 | 3.896 | 3.813 | >0.9999 |  |
|  |  | 20 | 4.001 | 3.713 | 0.2579 |  |
|  |  | 25 | 3.936 | 3.716 | 0.7319 |  |
|  |  | 30 | 3.781 | 3.674 | >0.9999 |  |
|  |  | 35 | 3.853 | 3.599 | 0.3169 |  |
|  |  | 40 | 3.843 | 3.625 | 0.4771 |  |
|  |  | 45 | 4.069 | 3.592 | 0.3723 |  |
|  |  | 50 | 4.055 | 3.568 | 0.3969 |  |
|  |  | 55 | 3.932 | 3.672 | 0.9334 |  |
|  |  | 60 | 3.987 | 3.64 | 0.7001 |  |
|  |  | 65 | 3.96 | 3.642 | 0.8494 |  |
|  |  | 70 | 3.817 | 3.646 | 0.9997 |  |
|  |  | 75 | 3.723 | 3.642 | >0.9999 |  |
|  |  | 80 | 3.711 | 3.609 | >0.9999 |  |
|  | **8** | 10 | 4.083 | 3.802 | 0.9714 |  |
|  |  | 15 | 3.946 | 3.809 | >0.9999 |  |
|  |  | 20 | 3.956 | 3.785 | 0.9947 |  |
|  |  | 25 | 3.987 | 3.852 | 0.9979 |  |
|  |  | 30 | 3.938 | 3.755 | 0.8919 |  |
|  |  | 35 | 3.855 | 3.718 | 0.9875 |  |
|  |  | 40 | 4.003 | 3.692 | 0.7364 |  |
|  |  | 45 | 3.999 | 3.748 | 0.6521 |  |
|  |  | 50 | 3.987 | 3.882 | >0.9999 |  |
|  |  | 55 | 4.016 | 3.733 | 0.6224 |  |
|  |  | 60 | 3.922 | 3.703 | 0.7777 |  |
|  |  | 65 | 3.881 | 3.688 | 0.8014 |  |
|  |  | 70 | 3.881 | 3.673 | 0.7969 |  |
|  |  | 75 | 3.858 | 3.662 | 0.6689 |  |
|  |  | 80 | 3.858 | 3.673 | 0.7861 |  |
|  | **12** | 25 | 4.055 | 3.778 | 0.0759 |  |
|  |  | 30 | 3.918 | 3.785 | 0.9998 |  |
|  |  | 35 | 3.937 | 3.714 | 0.4708 |  |
|  |  | 40 | 4.102 | 3.744 | 0.5522 |  |
|  |  | 45 | 4.157 | 3.759 | 0.2038 |  |
|  |  | 50 | 4.004 | 3.761 | 0.614 |  |
|  |  | 55 | 4.016 | 3.74 | 0.482 |  |
|  |  | 60 | 3.975 | 3.692 | 0.4462 |  |
|  |  | 65 | 3.881 | 3.669 | 0.7391 |  |
|  |  | 70 | 3.922 | 3.658 | 0.5379 |  |
|  |  | 75 | 3.846 | 3.628 | 0.6526 |  |
|  |  | 80 | 3.887 | 3.628 | 0.4827 |  |
|  | **16** | 35 | 4.336 | 3.781 | 0.0064 |  |
|  |  | 40 | 4.174 | 3.8 | 0.1631 |  |
|  |  | 45 | 4.245 | 3.751 | 0.0304 |  |
|  |  | 50 | 4.281 | 3.759 | 0.0777 |  |
|  |  | 55 | 4.157 | 3.718 | 0.1344 |  |
|  |  | 60 | 4.063 | 3.673 | 0.1751 |  |
|  |  | 65 | 4.022 | 3.729 | 0.3667 |  |
|  |  | 70 | 3.969 | 3.673 | 0.4222 |  |
|  |  | 75 | 3.946 | 3.677 | 0.3891 |  |
|  |  | 80 | 3.905 | 3.62 | 0.3565 |  |
|  | **24** | 45 | 4.162 | 4.094 | >0.9999 |  |
|  |  | 50 | 4.151 | 3.966 | 0.9317 |  |
|  |  | 55 | 4.104 | 3.849 | 0.561 |  |
|  |  | 60 | 3.993 | 3.763 | 0.5355 |  |
|  |  | 65 | 3.963 | 3.744 | 0.7812 |  |
|  |  | 70 | 3.916 | 3.755 | 0.9262 |  |
|  |  | 75 | 3.887 | 3.725 | 0.9453 |  |
|  |  | 80 | 3.869 | 3.714 | 0.9466 |  |
|  | **32** | 55 | 4.077 | 3.991 | 0.997 |  |
|  |  | 60 | 4.045 | 3.957 | 0.9942 |  |
|  |  | 65 | 4.028 | 3.882 | 0.9674 |  |
|  |  | 70 | 3.91 | 3.905 | >0.9999 |  |
|  |  | 75 | 3.869 | 3.781 | 0.9932 |  |
|  |  | 80 | 3.687 | 3.753 | 0.9993 |  |
| **4** | **4** | 10 | 5.117 | 5.025 | >0.9999 |  |
|  |  | 15 | 5.047 | 4.982 | >0.9999 |  |
|  |  | 20 | 5.014 | 4.887 | >0.9999 |  |
|  |  | 25 | 4.974 | 4.834 | 0.9996 |  |
|  |  | 30 | 4.953 | 4.817 | >0.9999 |  |
|  |  | 35 | 4.912 | 4.749 | 0.9998 |  |
|  |  | 40 | 4.965 | 4.714 | 0.9616 |  |
|  |  | 45 | 4.997 | 4.669 | 0.5248 |  |
|  |  | 50 | 4.939 | 4.636 | 0.7197 |  |
|  |  | 55 | 4.857 | 4.553 | 0.7234 |  |
|  |  | 60 | 4.909 | 4.608 | 0.8476 |  |
|  |  | 65 | 4.823 | 4.632 | 0.9992 |  |
|  |  | 70 | 4.738 | 4.682 | >0.9999 |  |
|  |  | 75 | 4.691 | 4.591 | >0.9999 |  |
|  |  | 80 | 4.562 | 4.538 | >0.9999 |  |
|  | **8** | 10 | 5.316 | 5.056 | 0.9939 |  |
|  |  | 15 | 5.22 | 5.037 | >0.9999 |  |
|  |  | 20 | 5.127 | 4.981 | >0.9999 |  |
|  |  | 25 | 5.162 | 4.921 | 0.8802 |  |
|  |  | 30 | 5.162 | 4.917 | 0.9373 |  |
|  |  | 35 | 5.064 | 4.85 | 0.9662 |  |
|  |  | 40 | 5.056 | 4.854 | 0.9894 |  |
|  |  | 45 | 5.014 | 4.712 | 0.6289 |  |
|  |  | 50 | 5.02 | 4.656 | 0.4795 |  |
|  |  | 55 | 4.938 | 4.689 | 0.9214 |  |
|  |  | 60 | 4.856 | 4.685 | 0.9976 |  |
|  |  | 65 | 4.809 | 4.622 | 0.9866 |  |
|  |  | 70 | 4.803 | 4.581 | 0.9471 |  |
|  |  | 75 | 4.756 | 4.57 | 0.9785 |  |
|  |  | 80 | 4.662 | 4.653 | >0.9999 |  |
|  | **12** | 25 | 5.192 | 4.932 | 0.8126 |  |
|  |  | 30 | 5.138 | 4.966 | 0.9897 |  |
|  |  | 35 | 5.072 | 4.895 | 0.8542 |  |
|  |  | 40 | 5.08 | 4.842 | 0.892 |  |
|  |  | 45 | 5.079 | 4.734 | 0.2669 |  |
|  |  | 50 | 5.008 | 4.706 | 0.4622 |  |
|  |  | 55 | 4.967 | 4.706 | 0.8041 |  |
|  |  | 60 | 4.903 | 4.622 | 0.8117 |  |
|  |  | 65 | 4.832 | 4.633 | 0.9824 |  |
|  |  | 70 | 4.803 | 4.562 | 0.908 |  |
|  |  | 75 | 4.791 | 4.612 | 0.9948 |  |
|  |  | 80 | 4.697 | 4.506 | 0.9776 |  |
|  | **16** | 35 | 5.107 | 4.928 | 0.8681 |  |
|  |  | 40 | 5.11 | 4.899 | 0.568 |  |
|  |  | 45 | 5.138 | 4.839 | 0.2642 |  |
|  |  | 50 | 5.085 | 4.794 | 0.2845 |  |
|  |  | 55 | 5.014 | 4.629 | 0.3719 |  |
|  |  | 60 | 4.985 | 4.714 | 0.5876 |  |
|  |  | 65 | 4.932 | 4.685 | 0.7306 |  |
|  |  | 70 | 4.909 | 4.611 | 0.6773 |  |
|  |  | 75 | 4.85 | 4.585 | 0.8394 |  |
|  |  | 80 | 4.744 | 4.514 | 0.8873 |  |
|  | **24** | 45 | 5.261 | 4.969 | 0.9651 |  |
|  |  | 50 | 5.202 | 4.834 | 0.2067 |  |
|  |  | 55 | 5.038 | 4.685 | 0.0636 |  |
|  |  | 60 | 4.961 | 4.715 | 0.6854 |  |
|  |  | 65 | 4.926 | 4.669 | 0.6588 |  |
|  |  | 70 | 4.879 | 4.673 | 0.8819 |  |
|  |  | 75 | 4.826 | 4.616 | 0.9211 |  |
|  |  | 80 | 4.809 | 4.547 | 0.7675 |  |
|  | **32** | 55 | 4.99 | 4.917 | 0.9998 |  |
|  |  | 60 | 5.026 | 4.861 | 0.9629 |  |
|  |  | 65 | 4.95 | 4.857 | 0.9967 |  |
|  |  | 70 | 4.909 | 4.779 | 0.973 |  |
|  |  | 75 | 4.891 | 4.771 | 0.9709 |  |
|  |  | 80 | 4.668 | 4.71 | >0.9999 |  |
| **Inter-peak latency** |  |  |  |  |  |  |
| **Peak** | **Frequency (kHz)** | **Intensity (dB SPL)** | **WT Mean (mV)** | **C1q KO Mean (mV)** | **P value** |  |
| **I-II** | **4** | 10 | 1.089 | 1.007 | 0.9833 |  |
|  |  | 15 | 1.069 | 0.9575 | 0.8615 |  |
|  |  | 20 | 1.069 | 0.9371 | 0.6327 |  |
|  |  | 25 | 1.069 | 0.9906 | 0.9554 |  |
|  |  | 30 | 1.077 | 1.007 | 0.982 |  |
|  |  | 35 | 1.093 | 0.9821 | 0.9511 |  |
|  |  | 40 | 1.098 | 0.9738 | 0.7761 |  |
|  |  | 45 | 1.174 | 0.9165 | 0.0529 |  |
|  |  | 50 | 1.121 | 0.9 | 0.5961 |  |
|  |  | 55 | 1.098 | 0.9906 | 0.9996 |  |
|  |  | 60 | 1.204 | 1.18 | >0.9999 |  |
|  |  | 65 | 1.226 | 1.151 | >0.9999 |  |
|  |  | 70 | 1.162 | 1.122 | >0.9999 |  |
|  |  | 75 | 1.139 | 1.139 | >0.9999 |  |
|  |  | 80 | 1.168 | 1.134 | >0.9999 |  |
|  | **8** | 10 | 1.164 | 0.9727 | 0.8765 |  |
|  |  | 15 | 1.096 | 0.9589 | 0.9815 |  |
|  |  | 20 | 1.085 | 1.007 | 0.9871 |  |
|  |  | 25 | 1.11 | 1.035 | 0.9939 |  |
|  |  | 30 | 1.134 | 1.013 | 0.3118 |  |
|  |  | 35 | 1.134 | 1.02 | 0.4625 |  |
|  |  | 40 | 1.062 | 0.9674 | 0.9038 |  |
|  |  | 45 | 1.121 | 1.012 | 0.9872 |  |
|  |  | 50 | 1.221 | 1.024 | 0.5368 |  |
|  |  | 55 | 1.18 | 1.08 | 0.9957 |  |
|  |  | 60 | 1.163 | 1.203 | >0.9999 |  |
|  |  | 65 | 1.169 | 1.117 | >0.9999 |  |
|  |  | 70 | 1.157 | 1.166 | >0.9999 |  |
|  |  | 75 | 1.157 | 1.099 | >0.9999 |  |
|  |  | 80 | 1.221 | 1.099 | 0.8807 |  |
|  | **12** | 25 | 1.082 | 0.9641 | 0.8818 |  |
|  |  | 30 | 1.069 | 0.9789 | 0.8661 |  |
|  |  | 35 | 1.069 | 1.009 | 0.9991 |  |
|  |  | 40 | 1.239 | 0.9975 | 0.3451 |  |
|  |  | 45 | 1.139 | 0.9938 | 0.7685 |  |
|  |  | 50 | 1.192 | 1.054 | 0.7174 |  |
|  |  | 55 | 1.204 | 1.199 | >0.9999 |  |
|  |  | 60 | 1.251 | 1.181 | 0.9868 |  |
|  |  | 65 | 1.251 | 1.162 | 0.9449 |  |
|  |  | 70 | 1.257 | 1.166 | 0.8511 |  |
|  |  | 75 | 1.239 | 1.151 | 0.8592 |  |
|  |  | 80 | 1.239 | 1.11 | 0.5284 |  |
|  | **16** | 35 | 1.11 | 0.9975 | 0.2022 |  |
|  |  | 40 | 1.133 | 0.9528 | 0.032 |  |
|  |  | 45 | 1.186 | 0.93 | 0.0457 |  |
|  |  | 50 | 1.145 | 1.027 | 0.8944 |  |
|  |  | 55 | 1.198 | 1.058 | 0.7914 |  |
|  |  | 60 | 1.221 | 1.084 | 0.9383 |  |
|  |  | 65 | 1.174 | 1.199 | >0.9999 |  |
|  |  | 70 | 1.151 | 1.188 | >0.9999 |  |
|  |  | 75 | 1.239 | 1.166 | 0.9581 |  |
|  |  | 80 | 1.221 | 1.143 | 0.9518 |  |
|  | **24** | 45 | 0.9245 | 0.974 | >0.9999 |  |
|  |  | 50 | 1.033 | 0.8386 | 0.7424 |  |
|  |  | 55 | 0.8631 | 0.7846 | 0.9871 |  |
|  |  | 60 | 0.8513 | 0.7325 | 0.781 |  |
|  |  | 65 | 0.9809 | 0.9823 | >0.9999 |  |
|  |  | 70 | 1.004 | 1.106 | 0.9984 |  |
|  |  | 75 | 1.01 | 1.143 | 0.9819 |  |
|  |  | 80 | 1.045 | 1.084 | >0.9999 |  |
|  | **32** | 55 | 0.9454 | 0.8593 | 0.9757 |  |
|  |  | 60 | 0.9809 | 0.9565 | >0.9999 |  |
|  |  | 65 | 0.922 | 0.8258 | 0.9768 |  |
|  |  | 70 | 0.9924 | 0.9788 | >0.9999 |  |
|  |  | 75 | 0.8984 | 1.061 | 0.7812 |  |
|  |  | 80 | 0.8573 | 0.7274 | 0.9998 |  |
| **II-III** | **4** | 10 | 0.7395 | 0.596 | 0.9381 |  |
|  |  | 15 | 0.6742 | 0.7307 | >0.9999 |  |
|  |  | 20 | 0.8907 | 0.7398 | 0.7432 |  |
|  |  | 25 | 0.9043 | 0.7069 | 0.4273 |  |
|  |  | 30 | 0.7808 | 0.6905 | >0.9999 |  |
|  |  | 35 | 0.863 | 0.6941 | 0.915 |  |
|  |  | 40 | 0.7805 | 0.7397 | >0.9999 |  |
|  |  | 45 | 0.9453 | 0.7973 | 0.9995 |  |
|  |  | 50 | 1.048 | 0.818 | 0.91 |  |
|  |  | 55 | 1.007 | 0.8858 | 0.9998 |  |
|  |  | 60 | 0.9569 | 0.6713 | 0.7731 |  |
|  |  | 65 | 0.9727 | 0.7316 | 0.756 |  |
|  |  | 70 | 0.922 | 0.789 | 0.9985 |  |
|  |  | 75 | 0.8693 | 0.8096 | >0.9999 |  |
|  |  | 80 | 0.8633 | 0.7932 | >0.9999 |  |
|  | **8** | 15 | 0.685 | 0.6167 | 0.9986 |  |
|  |  | 20 | 0.6577 | 0.6759 | >0.9999 |  |
|  |  | 25 | 0.7193 | 0.6699 | >0.9999 |  |
|  |  | 30 | 0.7726 | 0.744 | >0.9999 |  |
|  |  | 35 | 0.74 | 0.6986 | >0.9999 |  |
|  |  | 40 | 0.707 | 0.7028 | >0.9999 |  |
|  |  | 45 | 0.9536 | 0.7646 | 0.8558 |  |
|  |  | 50 | 0.9277 | 0.8346 | 0.997 |  |
|  |  | 55 | 0.8749 | 0.933 | >0.9999 |  |
|  |  | 60 | 0.9687 | 0.7934 | 0.9849 |  |
|  |  | 65 | 0.951 | 0.6987 | 0.284 |  |
|  |  | 70 | 0.9396 | 0.8139 | 0.9702 |  |
|  |  | 75 | 0.9923 | 0.7808 | 0.3453 |  |
|  |  | 80 | 0.9281 | 0.8587 | 0.9974 |  |
|  | **12** | 25 | 0.8907 | 0.789 | 0.9267 |  |
|  |  | 30 | 0.822 | 0.8178 | >0.9999 |  |
|  |  | 35 | 0.8136 | 0.7768 | >0.9999 |  |
|  |  | 40 | 1.003 | 0.8509 | 0.9722 |  |
|  |  | 45 | 1.086 | 0.888 | 0.7874 |  |
|  |  | 50 | 0.9217 | 0.8631 | >0.9999 |  |
|  |  | 55 | 0.9744 | 0.708 | 0.1553 |  |
|  |  | 60 | 0.9217 | 0.7234 | 0.078 |  |
|  |  | 65 | 0.8806 | 0.7561 | 0.2641 |  |
|  |  | 70 | 0.9511 | 0.7685 | 0.1348 |  |
|  |  | 75 | 0.916 | 0.7684 | 0.1834 |  |
|  |  | 80 | 0.9629 | 0.7725 | 0.1193 |  |
|  | **16** | 35 | 1.161 | 0.8015 | 0.2099 |  |
|  |  | 40 | 1.015 | 0.9165 | 0.9977 |  |
|  |  | 45 | 1.098 | 0.9333 | 0.574 |  |
|  |  | 50 | 1.216 | 0.8429 | 0.1368 |  |
|  |  | 55 | 1.074 | 0.8057 | 0.4943 |  |
|  |  | 60 | 1.022 | 0.7645 | 0.3273 |  |
|  |  | 65 | 1.051 | 0.7563 | 0.1235 |  |
|  |  | 70 | 1.045 | 0.7314 | 0.1741 |  |
|  |  | 75 | 0.9746 | 0.7849 | 0.0577 |  |
|  |  | 80 | 0.9923 | 0.7806 | 0.0728 |  |
|  | **24** | 45 | 1.172 | 0.9317 | 0.9191 |  |
|  |  | 50 | 1.051 | 1.205 | 0.9661 |  |
|  |  | 55 | 1.368 | 1.249 | 0.9792 |  |
|  |  | 60 | 1.321 | 1.249 | 0.9979 |  |
|  |  | 65 | 1.186 | 1.046 | 0.9884 |  |
|  |  | 70 | 1.139 | 0.8838 | 0.6794 |  |
|  |  | 75 | 1.098 | 0.8222 | 0.6786 |  |
|  |  | 80 | 1.086 | 0.8796 | 0.6697 |  |
|  | **32** | 55 | 1.143 | 1.048 | 0.976 |  |
|  |  | 60 | 1.051 | 0.9534 | 0.9899 |  |
|  |  | 65 | 1.133 | 1.044 | 0.9929 |  |
|  |  | 70 | 1.063 | 0.9455 | 0.9744 |  |
|  |  | 75 | 1.116 | 0.8509 | 0.223 |  |
|  |  | 80 | 1.057 | 0.8586 | 0.6588 |  |
| **III-IV** | **4** | 10 | 1.059 | 1.233 | 0.8709 |  |
|  |  | 15 | 1.151 | 1.169 | >0.9999 |  |
|  |  | 20 | 1.014 | 1.174 | 0.9812 |  |
|  |  | 25 | 1.038 | 1.118 | >0.9999 |  |
|  |  | 30 | 1.171 | 1.142 | >0.9999 |  |
|  |  | 35 | 1.058 | 1.151 | >0.9999 |  |
|  |  | 40 | 1.126 | 1.089 | >0.9999 |  |
|  |  | 45 | 0.9276 | 1.077 | 0.8805 |  |
|  |  | 50 | 0.8837 | 1.068 | 0.8497 |  |
|  |  | 55 | 0.9248 | 0.8814 | >0.9999 |  |
|  |  | 60 | 0.922 | 0.9683 | >0.9999 |  |
|  |  | 65 | 0.8633 | 0.9905 | 0.9818 |  |
|  |  | 70 | 0.9217 | 1.036 | 0.9952 |  |
|  |  | 75 | 0.9686 | 0.9494 | >0.9999 |  |
|  |  | 80 | 0.904 | 0.9291 | >0.9999 |  |
|  | **8** | 15 | 1.274 | 1.228 | >0.9999 |  |
|  |  | 20 | 1.171 | 1.196 | >0.9999 |  |
|  |  | 25 | 1.176 | 1.064 | 0.9999 |  |
|  |  | 30 | 1.225 | 1.151 | >0.9999 |  |
|  |  | 35 | 1.208 | 1.122 | 0.9998 |  |
|  |  | 40 | 1.151 | 1.151 | >0.9999 |  |
|  |  | 45 | 1.016 | 0.9453 | 0.9997 |  |
|  |  | 50 | 1.033 | 0.8097 | 0.4767 |  |
|  |  | 55 | 0.9219 | 0.9616 | >0.9999 |  |
|  |  | 60 | 0.9336 | 0.9905 | >0.9999 |  |
|  |  | 65 | 0.9274 | 0.9657 | >0.9999 |  |
|  |  | 70 | 0.9217 | 0.9413 | >0.9999 |  |
|  |  | 75 | 0.898 | 0.9819 | 0.997 |  |
|  |  | 80 | 0.804 | 0.9821 | 0.8836 |  |
|  | **12** | 25 | 1.137 | 1.155 | >0.9999 |  |
|  |  | 30 | 1.219 | 1.18 | >0.9999 |  |
|  |  | 35 | 1.135 | 1.192 | >0.9999 |  |
|  |  | 40 | 0.978 | 1.097 | 0.9269 |  |
|  |  | 45 | 0.9221 | 0.9574 | >0.9999 |  |
|  |  | 50 | 1.004 | 0.9636 | 0.9998 |  |
|  |  | 55 | 0.9513 | 0.9726 | >0.9999 |  |
|  |  | 60 | 0.9279 | 0.9369 | >0.9999 |  |
|  |  | 65 | 0.9511 | 0.9989 | >0.9999 |  |
|  |  | 70 | 0.8806 | 0.9364 | >0.9999 |  |
|  |  | 75 | 0.9453 | 1.019 | 0.9999 |  |
|  |  | 80 | 0.8103 | 0.9084 | 0.9974 |  |
|  | **16** | 35 | 0.7708 | 1.192 | 0.0973 |  |
|  |  | 40 | 1 | 1.102 | 0.9533 |  |
|  |  | 45 | 0.8924 | 1.093 | 0.5396 |  |
|  |  | 50 | 0.8043 | 1.068 | 0.4742 |  |
|  |  | 55 | 0.8573 | 0.9245 | 0.9974 |  |
|  |  | 60 | 0.9216 | 0.9863 | 0.9911 |  |
|  |  | 65 | 0.9099 | 0.9534 | 0.9983 |  |
|  |  | 70 | 0.9394 | 0.9331 | >0.9999 |  |
|  |  | 75 | 0.9039 | 0.9004 | >0.9999 |  |
|  |  | 80 | 0.8396 | 0.9251 | 0.9888 |  |
|  | **24** | 45 | 1.1 | 0.9198 | 0.9922 |  |
|  |  | 50 | 1.051 | 0.8907 | 0.9183 |  |
|  |  | 55 | 0.9339 | 0.8465 | 0.9462 |  |
|  |  | 60 | 0.9686 | 0.9698 | >0.9999 |  |
|  |  | 65 | 0.9629 | 0.9407 | >0.9999 |  |
|  |  | 70 | 0.963 | 0.8812 | 0.9314 |  |
|  |  | 75 | 0.9393 | 0.8629 | 0.9751 |  |
|  |  | 80 | 0.9393 | 0.8547 | 0.9623 |  |
|  | **32** | 55 | 0.9124 | 0.9 | >0.9999 |  |
|  |  | 60 | 0.9809 | 0.929 | 0.9991 |  |
|  |  | 65 | 0.922 | 1.011 | 0.9287 |  |
|  |  | 70 | 0.9981 | 0.9205 | 0.9627 |  |
|  |  | 75 | 1.021 | 1.007 | >0.9999 |  |
|  |  | 80 | 0.9804 | 0.9453 | 0.9997 |  |
| **I-III** | **4** | 10 | 1.829 | 1.603 | 0.9305 |  |
|  |  | 15 | 1.743 | 1.671 | >0.9999 |  |
|  |  | 20 | 1.959 | 1.662 | 0.0652 |  |
|  |  | 25 | 1.942 | 1.698 | 0.2138 |  |
|  |  | 30 | 1.839 | 1.697 | >0.9999 |  |
|  |  | 35 | 1.942 | 1.667 | 0.095 |  |
|  |  | 40 | 1.863 | 1.714 | 0.8181 |  |
|  |  | 45 | 2.12 | 1.714 | 0.3273 |  |
|  |  | 50 | 2.151 | 1.718 | 0.3313 |  |
|  |  | 55 | 2.069 | 1.872 | 0.8806 |  |
|  |  | 60 | 2.161 | 1.872 | 0.601 |  |
|  |  | 65 | 2.199 | 1.883 | 0.5739 |  |
|  |  | 70 | 2.084 | 1.911 | 0.9992 |  |
|  |  | 75 | 2.008 | 1.948 | >0.9999 |  |
|  |  | 80 | 2.032 | 1.927 | >0.9999 |  |
|  | **8** | 10 | 1.849 | 1.589 | 0.9444 |  |
|  |  | 15 | 1.754 | 1.635 | >0.9999 |  |
|  |  | 20 | 1.788 | 1.677 | 0.9999 |  |
|  |  | 25 | 1.882 | 1.751 | 0.999 |  |
|  |  | 30 | 1.874 | 1.693 | 0.8925 |  |
|  |  | 35 | 1.841 | 1.702 | 0.9806 |  |
|  |  | 40 | 2.006 | 1.714 | 0.7215 |  |
|  |  | 45 | 2.049 | 1.804 | 0.4852 |  |
|  |  | 50 | 2.096 | 1.928 | 0.8606 |  |
|  |  | 55 | 2.149 | 1.85 | 0.1802 |  |
|  |  | 60 | 2.114 | 1.887 | 0.301 |  |
|  |  | 65 | 2.108 | 1.907 | 0.4136 |  |
|  |  | 70 | 2.149 | 1.928 | 0.2222 |  |
|  |  | 75 | 2.138 | 1.919 | 0.0749 |  |
|  |  | 80 | 2.149 | 1.936 | 0.1199 |  |
|  | **12** | 25 | 3.11 | 2.889 | 0.721 |  |
|  |  | 30 | 3.11 | 2.955 | 0.9758 |  |
|  |  | 35 | 3.017 | 2.935 | 0.9987 |  |
|  |  | 40 | 3.132 | 2.906 | 0.8596 |  |
|  |  | 45 | 3.147 | 2.807 | 0.1244 |  |
|  |  | 50 | 3.118 | 2.845 | 0.291 |  |
|  |  | 55 | 3.13 | 2.886 | 0.6058 |  |
|  |  | 60 | 3.1 | 2.828 | 0.6758 |  |
|  |  | 65 | 3.083 | 2.898 | 0.957 |  |
|  |  | 70 | 3.088 | 2.877 | 0.8542 |  |
|  |  | 75 | 3.1 | 2.946 | 0.9953 |  |
|  |  | 80 | 3.012 | 2.774 | 0.7978 |  |
|  | **16** | 35 | 2.271 | 1.776 | 0.0256 |  |
|  |  | 40 | 2.149 | 1.85 | 0.4278 |  |
|  |  | 45 | 2.284 | 1.841 | 0.0246 |  |
|  |  | 50 | 2.36 | 1.842 | 0.0408 |  |
|  |  | 55 | 2.272 | 1.837 | 0.067 |  |
|  |  | 60 | 2.243 | 1.825 | 0.0558 |  |
|  |  | 65 | 2.225 | 1.936 | 0.1085 |  |
|  |  | 70 | 2.196 | 1.903 | 0.1371 |  |
|  |  | 75 | 2.214 | 1.936 | 0.0817 |  |
|  |  | 80 | 2.214 | 1.899 | 0.0614 |  |
|  | **24** | 45 | 2.096 | 1.877 | 0.9515 |  |
|  |  | 50 | 2.084 | 1.986 | 0.9953 |  |
|  |  | 55 | 2.231 | 1.977 | 0.5342 |  |
|  |  | 60 | 2.173 | 1.928 | 0.2851 |  |
|  |  | 65 | 2.167 | 1.95 | 0.5824 |  |
|  |  | 70 | 2.143 | 1.94 | 0.7952 |  |
|  |  | 75 | 2.108 | 1.94 | 0.9084 |  |
|  |  | 80 | 2.132 | 1.944 | 0.7775 |  |
|  | **32** | 55 | 2.088 | 1.849 | 0.0823 |  |
|  |  | 60 | 2.032 | 1.866 | 0.4657 |  |
|  |  | 65 | 2.055 | 1.825 | 0.4695 |  |
|  |  | 70 | 2.055 | 1.899 | 0.678 |  |
|  |  | 75 | 2.014 | 1.874 | 0.8737 |  |
|  |  | 80 | 1.914 | 1.941 | >0.9999 |  |
| **I-IV** | **4** | 10 | 2.887 | 2.836 | >0.9999 |  |
|  |  | 15 | 2.894 | 2.841 | >0.9999 |  |
|  |  | 20 | 2.973 | 2.836 | 0.997 |  |
|  |  | 25 | 2.98 | 2.815 | 0.9951 |  |
|  |  | 30 | 3.01 | 2.84 | 0.9988 |  |
|  |  | 35 | 3 | 2.817 | 0.9971 |  |
|  |  | 40 | 2.979 | 2.803 | 0.9988 |  |
|  |  | 45 | 3.047 | 2.791 | 0.4916 |  |
|  |  | 50 | 3.035 | 2.786 | 0.6853 |  |
|  |  | 55 | 2.994 | 2.754 | 0.6454 |  |
|  |  | 60 | 3.083 | 2.841 | 0.8772 |  |
|  |  | 65 | 3.062 | 2.873 | 0.9903 |  |
|  |  | 70 | 3.006 | 2.947 | >0.9999 |  |
|  |  | 75 | 2.977 | 2.898 | >0.9999 |  |
|  |  | 80 | 2.088 | 2.857 | 0.9994 |  |
|  | **8** | 10 | 3.082 | 2.843 | 0.9863 |  |
|  |  | 15 | 3.028 | 2.863 | >0.9999 |  |
|  |  | 20 | 2.959 | 2.873 | >0.9999 |  |
|  |  | 25 | 3.058 | 2.815 | 0.8144 |  |
|  |  | 30 | 3.099 | 2.844 | 0.8463 |  |
|  |  | 35 | 3.05 | 2.823 | 0.8546 |  |
|  |  | 40 | 3.072 | 2.865 | 0.9637 |  |
|  |  | 45 | 3.065 | 2.75 | 0.3214 |  |
|  |  | 50 | 3.13 | 2.737 | 0.1191 |  |
|  |  | 55 | 3.071 | 2.811 | 0.7672 |  |
|  |  | 60 | 3.047 | 2.877 | 0.9902 |  |
|  |  | 65 | 3.036 | 2.873 | 0.9936 |  |
|  |  | 70 | 3.071 | 2.869 | 0.8945 |  |
|  |  | 75 | 3.036 | 2.296 | 0.9849 |  |
|  |  | 80 | 2.953 | 2.358 | 0.9982 |  |
|  | **12** | 25 | 3.11 | 2.889 | 0.721 |  |
|  |  | 30 | 3.11 | 2.955 | 0.9758 |  |
|  |  | 35 | 3.017 | 2.935 | 0.9987 |  |
|  |  | 40 | 3.132 | 2.906 | 0.8596 |  |
|  |  | 45 | 3.147 | 2.807 | 0.1244 |  |
|  |  | 50 | 3.118 | 2.845 | 0.291 |  |
|  |  | 55 | 3.13 | 2.886 | 0.6058 |  |
|  |  | 60 | 3.1 | 2.828 | 0.6758 |  |
|  |  | 65 | 3.083 | 2.898 | 0.957 |  |
|  |  | 70 | 3.088 | 2.877 | 0.8542 |  |
|  |  | 75 | 3.1 | 2.946 | 0.9953 |  |
|  |  | 80 | 3.012 | 2.774 | 0.7978 |  |
|  | **16** | 35 | 3.042 | 2.968 | 0.9996 |  |
|  |  | 40 | 3.076 | 2.951 | 0.9545 |  |
|  |  | 45 | 3.176 | 2.934 | 0.3473 |  |
|  |  | 50 | 3.165 | 2.91 | 0.2665 |  |
|  |  | 55 | 3.129 | 2.762 | 0.4506 |  |
|  |  | 60 | 3.165 | 2.309 | 0.8655 |  |
|  |  | 65 | 3.135 | 2.889 | 0.4248 |  |
|  |  | 70 | 3.136 | 2.836 | 0.4521 |  |
|  |  | 75 | 3.118 | 2.836 | 0.5871 |  |
|  |  | 80 | 3.053 | 2.824 | 0.7811 |  |
|  | **24** | 45 | 3.196 | 2.749 | 0.2597 |  |
|  |  | 50 | 3.135 | 2.877 | 0.6601 |  |
|  |  | 55 | 3.165 | 2.823 | 0.0801 |  |
|  |  | 60 | 3.141 | 2.897 | 0.4335 |  |
|  |  | 65 | 3.13 | 2.891 | 0.5137 |  |
|  |  | 70 | 3.106 | 2.9 | 0.7259 |  |
|  |  | 75 | 3.047 | 2.877 | 0.9296 |  |
|  |  | 80 | 3.071 | 2.799 | 0.4925 |  |
|  | **32** | 55 | 3 | 2.749 | 0.5228 |  |
|  |  | 60 | 3.012 | 2.795 | 0.554 |  |
|  |  | 65 | 2.977 | 2.836 | 0.8637 |  |
|  |  | 70 | 3.053 | 2.819 | 0.2805 |  |
|  |  | 75 | 3.035 | 2.881 | 0.7638 |  |
|  |  | 80 | 2.895 | 2.886 | >0.9999 |  |
| **Amplitude** |  |  |  |  |  |  |
| **Peak** | **Frequency (kHz)** | **Intensity (dB SPL)** | **WT Mean (nV)** | **C1q KO Mean (nV)** | **P value** |  |
| **1** | **4** | 10 | 595.2 | 682.6 | >0.9999 |  |
|  |  | 15 | 1145 | 788.6 | 0.3148 |  |
|  |  | 20 | 1218 | 1095 | >0.9999 |  |
|  |  | 25 | 1396 | 1298 | >0.9999 |  |
|  |  | 30 | 1577 | 1401 | >0.9999 |  |
|  |  | 35 | 1589 | 1560 | >0.9999 |  |
|  |  | 40 | 1579 | 1675 | >0.9999 |  |
|  |  | 45 | 1682 | 1989 | >0.9999 |  |
|  |  | 50 | 1867 | 2178 | >0.9999 |  |
|  |  | 55 | 2104 | 2537 | 0.9985 |  |
|  |  | 60 | 2451 | 2763 | >0.9999 |  |
|  |  | 65 | 2890 | 3038 | >0.9999 |  |
|  |  | 70 | 3557 | 3445 | >0.9999 |  |
|  |  | 75 | 3949 | 3843 | >0.9999 |  |
|  |  | 80 | 4309 | 4186 | >0.9999 |  |
|  | **8** | 10 | 492.6 | 448.1 | >0.9999 |  |
|  |  | 15 | 874.2 | 548.8 | 0.9975 |  |
|  |  | 20 | 775.4 | 656.8 | >0.9999 |  |
|  |  | 25 | 813.7 | 879.3 | >0.9999 |  |
|  |  | 30 | 988.2 | 1020 | >0.9999 |  |
|  |  | 35 | 1223 | 1083 | >0.9999 |  |
|  |  | 40 | 1128 | 1263 | >0.9999 |  |
|  |  | 45 | 1402 | 1618 | >0.9999 |  |
|  |  | 50 | 1543 | 1943 | 0.995 |  |
|  |  | 55 | 2025 | 2193 | >0.9999 |  |
|  |  | 60 | 2518 | 2614 | >0.9999 |  |
|  |  | 65 | 3201 | 3082 | >0.9999 |  |
|  |  | 70 | 3464 | 3458 | >0.9999 |  |
|  |  | 75 | 3818 | 3747 | >0.9999 |  |
|  |  | 80 | 4067 | 4092 | >0.9999 |  |
|  | **12** | 25 | 983.2 | 919 | >0.9999 |  |
|  |  | 30 | 1141 | 1121 | >0.9999 |  |
|  |  | 35 | 1323 | 1245 | >0.9999 |  |
|  |  | 40 | 1108 | 1582 | 0.8922 |  |
|  |  | 45 | 1337 | 1902 | 0.8344 |  |
|  |  | 50 | 1712 | 2107 | 0.9901 |  |
|  |  | 55 | 2033 | 2477 | 0.995 |  |
|  |  | 60 | 2312 | 2726 | 0.9986 |  |
|  |  | 65 | 2862 | 3204 | >0.9999 |  |
|  |  | 70 | 3197 | 3555 | >0.9999 |  |
|  |  | 75 | 3718 | 3908 | >0.9999 |  |
|  |  | 80 | 3992 | 4425 | >0.9999 |  |
|  | **16** | 35 | 757 | 1070 | 0.9874 |  |
|  |  | 40 | 829.4 | 1369 | 0.6706 |  |
|  |  | 45 | 1020 | 1700 | 0.5341 |  |
|  |  | 50 | 1224 | 1907 | 0.5253 |  |
|  |  | 55 | 1767 | 2127 | 0.9938 |  |
|  |  | 60 | 1942 | 2458 | 0.9596 |  |
|  |  | 65 | 2223 | 2707 | 0.9843 |  |
|  |  | 70 | 2867 | 3020 | >0.9999 |  |
|  |  | 75 | 3311 | 3496 | >0.9999 |  |
|  |  | 80 | 3983 | 3947 | >0.9999 |  |
|  | **24** | 45 | 520.6 | 680.4 | 0.9156 |  |
|  |  | 50 | 746.8 | 979 | 0.809 |  |
|  |  | 55 | 854.4 | 1335 | 0.5855 |  |
|  |  | 60 | 1135 | 1643 | 0.7037 |  |
|  |  | 65 | 1372 | 2009 | 0.4864 |  |
|  |  | 70 | 1714 | 2175 | 0.7943 |  |
|  |  | 75 | 1993 | 2497 | 0.7965 |  |
|  |  | 80 | 2525 | 2924 | 0.9674 |  |
|  | **32** | 55 | 788.9 | 1088 | 0.8815 |  |
|  |  | 60 | 870.8 | 1311 | 0.712 |  |
|  |  | 65 | 1063 | 1554 | 0.7365 |  |
|  |  | 70 | 1255 | 1673 | 0.795 |  |
|  |  | 75 | 1610 | 2055 | 0.8102 |  |
|  |  | 80 | 1974 | 2361 | 0.9292 |  |
| **2** | **4** | 10 | 1107 | 986.8 | >0.9999 |  |
|  |  | 15 | 1434 | 1080 | 0.5591 |  |
|  |  | 20 | 1475 | 1408 | >0.9999 |  |
|  |  | 25 | 1925 | 1554 | 0.9888 |  |
|  |  | 30 | 2250 | 1723 | 0.7037 |  |
|  |  | 35 | 2176 | 2008 | >0.9999 |  |
|  |  | 40 | 2105 | 2194 | >0.9999 |  |
|  |  | 45 | 2088 | 2004 | >0.9999 |  |
|  |  | 50 | 2341 | 1548 | 0.4296 |  |
|  |  | 55 | 2663 | 1563 | 0.0528 |  |
|  |  | 60 | 2819 | 2431 | 0.9977 |  |
|  |  | 65 | 3150 | 2701 | >0.9999 |  |
|  |  | 70 | 4020 | 3049 | 0.9859 |  |
|  |  | 75 | 4217 | 3340 | 0.9995 |  |
|  |  | 80 | 4287 | 3510 | >0.9999 |  |
|  | **8** | 10 | 764.1 | 674.5 | >0.9999 |  |
|  |  | 15 | 986.1 | 659.6 | 0.8577 |  |
|  |  | 20 | 1145 | 805.3 | 0.5632 |  |
|  |  | 25 | 1275 | 1163 | >0.9999 |  |
|  |  | 30 | 1590 | 1340 | 0.9996 |  |
|  |  | 35 | 1876 | 1447 | 0.6347 |  |
|  |  | 40 | 1976 | 1687 | 0.9998 |  |
|  |  | 45 | 1909 | 1473 | 0.9918 |  |
|  |  | 50 | 2350 | 1641 | 0.3894 |  |
|  |  | 55 | 3198 | 1967 | 0.2326 |  |
|  |  | 60 | 3447 | 2406 | 0.7483 |  |
|  |  | 65 | 3710 | 3296 | >0.9999 |  |
|  |  | 70 | 4456 | 3169 | 0.8131 |  |
|  |  | 75 | 4532 | 3617 | 0.9943 |  |
|  |  | 80 | 4996 | 3495 | 0.6512 |  |
|  | **12** | 25 | 1514 | 1228 | 0.9794 |  |
|  |  | 30 | 1721 | 1368 | 0.9979 |  |
|  |  | 35 | 1597 | 1594 | >0.9999 |  |
|  |  | 40 | 1936 | 1999 | >0.9999 |  |
|  |  | 45 | 2046 | 2029 | >0.9999 |  |
|  |  | 50 | 2479 | 1938 | 0.7984 |  |
|  |  | 55 | 2906 | 2266 | 0.7269 |  |
|  |  | 60 | 3615 | 2682 | 0.8614 |  |
|  |  | 65 | 3669 | 3743 | >0.9999 |  |
|  |  | 70 | 4350 | 4122 | >0.9999 |  |
|  |  | 75 | 4995 | 4980 | >0.9999 |  |
|  |  | 80 | 5823 | 5093 | 0.9998 |  |
|  | **16** | 35 | 1470 | 1633 | >0.9999 |  |
|  |  | 40 | 1576 | 1860 | 0.9936 |  |
|  |  | 45 | 1928 | 2022 | >0.9999 |  |
|  |  | 50 | 2175 | 1983 | 0.9982 |  |
|  |  | 55 | 2670 | 2171 | 0.8763 |  |
|  |  | 60 | 3181 | 2452 | 0.6612 |  |
|  |  | 65 | 3480 | 3273 | >0.9999 |  |
|  |  | 70 | 4324 | 4407 | >0.9999 |  |
|  |  | 75 | 4919 | 4657 | >0.9999 |  |
|  |  | 80 | 5653 | 5140 | 0.9999 |  |
|  | **24** | 45 | 1044 | 1300 | 0.9696 |  |
|  |  | 50 | 1145 | 1359 | 0.9821 |  |
|  |  | 55 | 1266 | 1534 | 0.9793 |  |
|  |  | 60 | 1206 | 1350 | 0.9996 |  |
|  |  | 65 | 1554 | 1491 | >0.9999 |  |
|  |  | 70 | 1698 | 1495 | 0.9966 |  |
|  |  | 75 | 2078 | 2385 | 0.9985 |  |
|  |  | 80 | 2115 | 2636 | 0.9722 |  |
|  | **32** | 55 | 1546 | 1233 | 0.9861 |  |
|  |  | 60 | 1669 | 1526 | 0.999 |  |
|  |  | 65 | 1728 | 1433 | 0.9237 |  |
|  |  | 70 | 1606 | 1646 | >0.9999 |  |
|  |  | 75 | 1392 | 1530 | 0.9996 |  |
|  |  | 80 | 1521 | 1785 | 0.9835 |  |
| **3** | **4** | 10 | 342.6 | 323.3 | >0.9999 |  |
|  |  | 15 | 386 | 557.8 | 0.999 |  |
|  |  | 20 | 622.5 | 649.5 | >0.9999 |  |
|  |  | 25 | 697 | 759.5 | >0.9999 |  |
|  |  | 30 | 1100 | 675.3 | 0.851 |  |
|  |  | 35 | 1100 | 957 | 0.9996 |  |
|  |  | 40 | 763.5 | 1072 | 0.9972 |  |
|  |  | 45 | 1263 | 1177 | >0.9999 |  |
|  |  | 50 | 1495 | 1453 | >0.9999 |  |
|  |  | 55 | 1614 | 1574 | >0.9999 |  |
|  |  | 60 | 2141 | 1713 | 0.994 |  |
|  |  | 65 | 2714 | 1902 | 0.9688 |  |
|  |  | 70 | 3192 | 2471 | 0.9952 |  |
|  |  | 75 | 3356 | 2855 | >0.9999 |  |
|  |  | 80 | 3351 | 2922 | >0.9999 |  |
|  | **8** | 10 | 315 | 287.4 | >0.9999 |  |
|  |  | 15 | 289.6 | 363 | >0.9999 |  |
|  |  | 20 | 480.3 | 490 | >0.9999 |  |
|  |  | 25 | 516.2 | 573.7 | >0.9999 |  |
|  |  | 30 | 539.9 | 771.4 | 0.9626 |  |
|  |  | 35 | 507.6 | 776.4 | 0.9875 |  |
|  |  | 40 | 904.7 | 913.3 | >0.9999 |  |
|  |  | 45 | 913.1 | 1156 | 0.9979 |  |
|  |  | 50 | 1571 | 1560 | >0.9999 |  |
|  |  | 55 | 2309 | 1935 | 0.9977 |  |
|  |  | 60 | 3096 | 2601 | 0.9998 |  |
|  |  | 65 | 3550 | 3485 | >0.9999 |  |
|  |  | 70 | 3797 | 3950 | >0.9999 |  |
|  |  | 75 | 4064 | 3777 | >0.9999 |  |
|  |  | 80 | 4010 | 3645 | >0.9999 |  |
|  | **12** | 25 | 357.7 | 598.8 | 0.9554 |  |
|  |  | 30 | 341.6 | 648.4 | 0.8545 |  |
|  |  | 35 | 358.4 | 701 | 0.4696 |  |
|  |  | 40 | 585.4 | 858.6 | 0.9466 |  |
|  |  | 45 | 952.8 | 1229 | 0.9966 |  |
|  |  | 50 | 1389 | 1839 | 0.9266 |  |
|  |  | 55 | 1939 | 2351 | 0.9972 |  |
|  |  | 60 | 2683 | 2744 | >0.9999 |  |
|  |  | 65 | 3224 | 3544 | >0.9999 |  |
|  |  | 70 | 3798 | 3882 | >0.9999 |  |
|  |  | 75 | 3977 | 3996 | >0.9999 |  |
|  |  | 80 | 3972 | 3805 | >0.9999 |  |
|  | **16** | 35 | 786.1 | 722.2 | >0.9999 |  |
|  |  | 40 | 489 | 827.3 | 0.5836 |  |
|  |  | 45 | 956.5 | 838.4 | 0.9993 |  |
|  |  | 50 | 1079 | 1039 | >0.9999 |  |
|  |  | 55 | 1662 | 1633 | >0.9999 |  |
|  |  | 60 | 2119 | 2206 | >0.9999 |  |
|  |  | 65 | 2613 | 2465 | >0.9999 |  |
|  |  | 70 | 3153 | 2820 | >0.9999 |  |
|  |  | 75 | 3595 | 3248 | >0.9999 |  |
|  |  | 80 | 3529 | 3539 | >0.9999 |  |
|  | **24** | 45 | 767.2 | 1032 | 0.9817 |  |
|  |  | 50 | 731.7 | 1267 | 0.139 |  |
|  |  | 55 | 1376 | 1866 | 0.4079 |  |
|  |  | 60 | 1649 | 2127 | 0.7883 |  |
|  |  | 65 | 1712 | 2138 | 0.812 |  |
|  |  | 70 | 1822 | 2012 | 0.9988 |  |
|  |  | 75 | 2093 | 2326 | 0.9911 |  |
|  |  | 80 | 2414 | 2615 | 0.9999 |  |
|  | **32** | 55 | 839.3 | 1015 | 0.9952 |  |
|  |  | 60 | 999.6 | 1326 | 0.9182 |  |
|  |  | 65 | 1322 | 1687 | 0.9642 |  |
|  |  | 70 | 1589 | 1953 | 0.9592 |  |
|  |  | 75 | 2378 | 2019 | 0.9842 |  |
|  |  | 80 | 2476 | 2499 | >0.9999 |  |
| **4** | **4** | 10 | 891.7 | 1625 | 0.3844 |  |
|  |  | 15 | 1167 | 1628 | 0.9342 |  |
|  |  | 20 | 1560 | 1978 | 0.9934 |  |
|  |  | 25 | 1482 | 2079 | 0.9 |  |
|  |  | 30 | 1865 | 2148 | >0.9999 |  |
|  |  | 35 | 1752 | 2332 | 0.9247 |  |
|  |  | 40 | 1862 | 2386 | 0.9902 |  |
|  |  | 45 | 1255 | 2399 | 0.3763 |  |
|  |  | 50 | 1493 | 2601 | 0.522 |  |
|  |  | 55 | 1436 | 2483 | 0.4531 |  |
|  |  | 60 | 1404 | 2299 | 0.6211 |  |
|  |  | 65 | 1716 | 1967 | >0.9999 |  |
|  |  | 70 | 1772 | 2130 | 0.9998 |  |
|  |  | 75 | 1985 | 2031 | >0.9999 |  |
|  |  | 80 | 1726 | 1707 | >0.9999 |  |
|  | **8** | 10 | 925.9 | 1177 | >0.9999 |  |
|  |  | 15 | 777.9 | 1173 | 0.9558 |  |
|  |  | 20 | 1013 | 1373 | 0.9673 |  |
|  |  | 25 | 1104 | 1339 | 0.9994 |  |
|  |  | 30 | 1330 | 1514 | >0.9999 |  |
|  |  | 35 | 1374 | 1731 | 0.9993 |  |
|  |  | 40 | 1663 | 2170 | 0.9612 |  |
|  |  | 45 | 1452 | 2195 | 0.8782 |  |
|  |  | 50 | 1536 | 2044 | 0.9975 |  |
|  |  | 55 | 1711 | 1943 | >0.9999 |  |
|  |  | 60 | 1811 | 2073 | >0.9999 |  |
|  |  | 65 | 1736 | 2126 | 0.9999 |  |
|  |  | 70 | 1855 | 2137 | >0.9999 |  |
|  |  | 75 | 1368 | 1981 | 0.9486 |  |
|  |  | 80 | 1159 | 1884 | 0.9363 |  |
|  | **12** | 25 | 1504 | 1671 | >0.9999 |  |
|  |  | 30 | 1698 | 1980 | >0.9999 |  |
|  |  | 35 | 1557 | 2269 | 0.7915 |  |
|  |  | 40 | 1641 | 2387 | 0.9107 |  |
|  |  | 45 | 1520 | 2360 | 0.7398 |  |
|  |  | 50 | 1525 | 2436 | 0.6811 |  |
|  |  | 55 | 1597 | 2268 | 0.9074 |  |
|  |  | 60 | 1888 | 2251 | 0.9993 |  |
|  |  | 65 | 1843 | 2172 | 0.9999 |  |
|  |  | 70 | 2130 | 2310 | >0.9999 |  |
|  |  | 75 | 2036 | 2214 | >0.9999 |  |
|  |  | 80 | 1405 | 1742 | 0.9985 |  |
|  | **16** | 35 | 1047 | 2084 | 0.4502 |  |
|  |  | 40 | 1305 | 2279 | 0.479 |  |
|  |  | 45 | 1561 | 2643 | 0.4403 |  |
|  |  | 50 | 1592 | 2617 | 0.6437 |  |
|  |  | 55 | 1785 | 2744 | 0.6857 |  |
|  |  | 60 | 1978 | 2737 | 0.857 |  |
|  |  | 65 | 1970 | 2635 | 0.9537 |  |
|  |  | 70 | 2091 | 2521 | 0.9974 |  |
|  |  | 75 | 1919 | 2173 | >0.9999 |  |
|  |  | 80 | 1459 | 1835 | 0.9968 |  |
|  | **24** | 45 | 1381 | 1986 | 0.9507 |  |
|  |  | 50 | 1222 | 1892 | 0.6312 |  |
|  |  | 55 | 1137 | 1898 | 0.3855 |  |
|  |  | 60 | 1326 | 1937 | 0.6992 |  |
|  |  | 65 | 1395 | 2096 | 0.4567 |  |
|  |  | 70 | 1727 | 2162 | 0.9547 |  |
|  |  | 75 | 1717 | 2074 | 0.9733 |  |
|  |  | 80 | 1716 | 1654 | >0.9999 |  |
|  | **32** | 55 | 1257 | 1868 | 0.8676 |  |
|  |  | 60 | 1420 | 1972 | 0.8424 |  |
|  |  | 65 | 1374 | 2146 | 0.5217 |  |
|  |  | 70 | 1568 | 2188 | 0.7707 |  |
|  |  | 75 | 1658 | 2083 | 0.9305 |  |
|  |  | 80 | 1667 | 2059 | 0.9266 |  |
